# Disentangling Mitochondria in Alzheimer’s Disease

**DOI:** 10.3390/ijms222111520

**Published:** 2021-10-26

**Authors:** Ashu Johri

**Affiliations:** Feil Family Brain and Mind Research Institute, Weill Cornell Medicine, New York, NY 10065, USA; johri.ashu@gmail.com

**Keywords:** Alzheimer’s disease, neurodegeneration, mitochondria, antioxidants, PGC-1α, sirtuins

## Abstract

Alzheimer’s disease (AD) is a major cause of dementia in older adults and is fast becoming a major societal and economic burden due to an increase in life expectancy. Age seems to be the major factor driving AD, and currently, only symptomatic treatments are available. AD has a complex etiology, although mitochondrial dysfunction, oxidative stress, inflammation, and metabolic abnormalities have been widely and deeply investigated as plausible mechanisms for its neuropathology. Aβ plaques and hyperphosphorylated tau aggregates, along with cognitive deficits and behavioral problems, are the hallmarks of the disease. Restoration of mitochondrial bioenergetics, prevention of oxidative stress, and diet and exercise seem to be effective in reducing Aβ and in ameliorating learning and memory problems. Many mitochondria-targeted antioxidants have been tested in AD and are currently in development. However, larger streamlined clinical studies are needed to provide hard evidence of benefits in AD. This review discusses the causative factors, as well as potential therapeutics employed in the treatment of AD.

## 1. Introduction

Alzheimer’s disease (AD) is an age-related progressive neurodegenerative disorder characterized by impairment of cognitive function, decline of memory, and behavioral and personality changes [1]. Studies have shown that the neuropathology of AD involves two neurodegenerative processes: the deposition of extracellular amyloid β-peptide (Aβ), i.e., amyloidogenesis, and the formation of intracellular tangles composed of hyper-phosphorylated tau protein, causing neurofibrillary degeneration [2]. Yet, the etiology of sporadic AD remains unclear. Treatments currently approved by the U.S. Food and Drug Administration (USFDA) include the cholinesterase inhibitors donepezil, galantamine, and rivastigmine, which have shown low central nervous system selectivity, and an N-methyl-D-aspartate (NMDA) receptor antagonist, memantine, which has shown limited beneficial effects in clinical trials [3,4]. The latest drug to be approved for the treatment of AD is Biogen’s (originally Neurimmune’s) aducanumab (Aduhelm), which is a monoclonal antibody that reduces amyloid plaques in the brain. All of these drugs have gastrointestinal side effects, and aducanumab is also known to cause bleeding in the brain. Other anti-Aβ immunotherapies are in development as well, but so far, none of the therapies tested has shown disease-modifying potential.

## 2. Mitochondrial Impairment in AD

**Figure 1:**
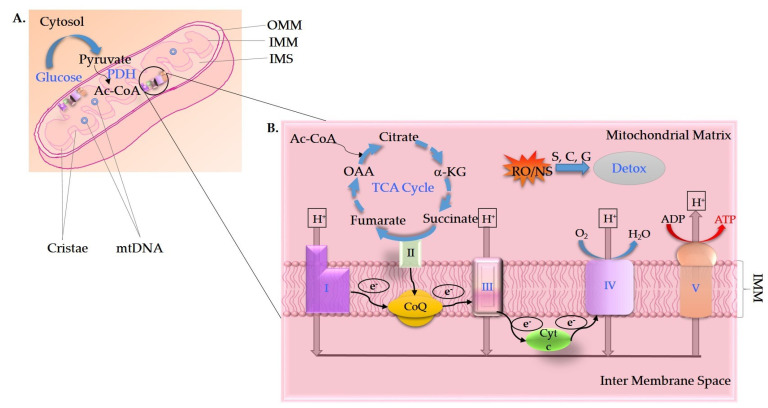
(**A**) Mitochondria are compartmentalized cellular organelles that contain an outer mitochondrial membrane (OMM), which is permeable to small molecules and proteins < 10 kD and contains porins (voltage-dependent anion channels (VDACs)), an inner mitochondrial membrane (IMM) that lacks nonspecific permeability, and an area between the OMM and IMM known as the intermembrane space (IMS). The inner membrane is highly folded into structures known as cristae, the site of ATP production. The space inside the IMM is filled with a gel-like mitochondrial matrix, which contains mitochondrial DNA (mtDNA), and enzymes of the tricarboxylic acid (TCA, also known as the citric acid or Krebs) cycle and fatty acid beta-oxidation, among others. (**B**) Glycolysis metabolizes glucose to pyruvate, which, after a series of reactions in the mitochondrial matrix, produces reducing equivalents nicotinamide adenine dinucleotide (NADH) and flavin adenine dinucleotide (FADH_2_) in the TCA cycle. The NADH and FADH_2_ are then re-oxidized in the electron transport chain (ETC). Mitochondrial cristae are the seat of mitochondrial oxidative phosphorylation (OXPHOS) machinery, namely ETC complexes I-IV, two electron carriers, and a specialized ATP-synthesizing enzyme called ATP synthase or complex V. The energy released by the transfer of electrons through ETC complexes is utilized to transport protons across the IMM into the IMS. The flux of protons back into the mitochondrial matrix is mostly mediated by ATP synthase, which harnesses the energy to generate ATP from ADP. Reactive oxygen/nitrogen species (RO/NS) are produced during this process, which are detoxified/countered (Detox) by antioxidant enzymes such as superoxide dismutase (S), catalase (C), and glutathione peroxidase (G), etc. Processes/molecules affected in AD are shown in bright blue color.

AD has recently been described as a multifactorial disease for which mitochondrial dysfunction lies at the forefront [5,6]. Along with mitochondrial dysfunction, several pathophysiological events, such as apoptosis, disruption of Ca^2+^ homeostasis, inflammation, oxidative stress, and deficient glucose metabolism, occur in the AD brain [7,8,9,10,11,12]. Mitochondrial dysfunction in AD is characterized by decreased activities of mitochondrial complex I (NADH:ubiquinone oxidoreductase), complex IV (cytochrome oxidase (COX)), complex V (ATPase), pyruvate dehydrogenase complex and α-ketoglutarate dehydrogenase complex, and increased reactive oxygen species (ROS) generation (Figure 1A,B) [13,14,15,16,17,18,19,20,21,22]. Activities of phosphofructokinase (PFK), phosphoglycerate mutase, aldolase, glucose-6-phosphate isomerase, and lactate dehydrogenase are also reduced in brain tissue samples of AD patients compared to age-matched controls [23]. Both mitochondrial numbers and morphology are affected in AD, the latter of which includes an increased amount of small and fragmented mitochondria [24,25,26,27,28]. A widely studied mouse model of AD, overexpressing mutant amyloid precursor protein (APP) in the brain, showed decreased mitochondrial membrane potential and respiration, increased mitochondrial ROS production and altered mitochondrial morphology, which preceded disease phenotypes [29,30,31,32,33,34]. Aβ impairs mitochondrial trafficking in neurons [35,36,37,38]. Expression and function of mitochondrial fission/fusion machinery is impaired in postmortem brains of AD patients, AD mouse models, and APP cell lines [39,40,41]. Proteomic and functional alterations in brain mitochondria from a transgenic mouse model of AD were shown to occur before overt plaque deposition [42]. Abnormal expression of mitochondrial-encoded genes was reported using quantitative real-time RT-PCR in different grades of AD postmortem brains compared to the brains of non-demented, healthy subjects, as well as in the blood of patients with early AD or mild cognitive impairment (MCI) [43,44]. These events may, in part, be caused by a direct effect of Aβ on mitochondria, since Aβ causes mitochondrial dysfunction when added to isolated mitochondria and in the primary cortical neurons of mice [30,45]. Rat brain mitochondria incubated with Aβ or isolated from Aβ-injected rat brains show decreased state 3 and 4 mitochondrial respiration, decreased activities of COX, α-ketoglutarate dehydrogenase and pyruvate dehydrogenase, ROS formation, mitochondrial membrane depolarization, mitochondrial swelling, cytochrome c release, and a significant decrease in the ATP/ADP ratio [30,46,47]. Deletion of mitochondrial ubiquitin ligase, which is involved in mitochondrial dynamics and functions and is dysregulated in AD, initiates mitochondrial impairments and worsens cognitive decline in a mouse model with AD-related Aβ pathology [48]. Other studies have examined the triggering of apoptotic cascades and organelle swelling following exposure of mitochondria to Aβ [49,50,51,52,53,54,55,56,57].

Increased intracellular Aβ levels may also facilitate mitochondrial permeability transition pore opening, a key event in cell death [58,59]. Intracellular Aβ progressively accumulates in mitochondria, aided by the translocase of the outer mitochondrial membrane, in the brains of transgenic mice with targeted neuronal overexpression of mutant human APP and is associated with diminished enzymatic activity of ETC complexes III and IV and a reduction in the rate of oxygen consumption. Moreover, mitochondria-associated Aβ42 was detected as early as 4 months of age, before extensive extracellular Aβ deposits. Interestingly, Aβ monomers and oligomers were shown to be associated with mitochondrial membranes in neurons from postmortem brain specimens of AD patients and mouse models of AD [60,61,62,63,64,65]. It is possible that Aβ directly interferes with the mitochondrial function and causes the metabolic deficiencies and neurological dysfunction observed in the brains of patients with AD [66]. For example, Aβ alters the physical and biochemical connections between the endoplasmic reticulum and mitochondria, as evidenced in AD brain and neuronal cultures, making the connections abnormally tight and interfering with mitochondrial morphology, motility, bioenergetics, autophagy, Ca^2+^ signaling, and apoptosis [67,68,69,70]. A direct membrane-binding of Aβ peptides was recently shown to block the mitochondrial large-conductance, Ca^2+^-activated potassium channels [71]. Further, C-terminal fragments of APP were shown to initiate mitochondrial structure, function, and mitophagy defects in various models of AD and postmortem sporadic AD brains [72]. Partial localization of tau and apolipoprotein E4 (apoE4) to mitochondria has also been noted [73,74]. C-terminal-truncated apoE4 has neurotoxic effects, and it alters mitochondrial respiratory function and causes mitochondrial Ca^2+^ overload by interfering with endoplasmic reticulum-mitochondrial-associated membrane function [75]. Overexpression of mutant tau decreases the activities of complexes I and V of the mitochondrial ETC [21,74]. Moreover, tau inhibits mitochondrial Ca^2+^ efflux via the mitochondrial Na^+^/Ca^2+^ exchanger and leads to mitochondrial depolarization in response to stimuli that induce Ca^2+^ signaling, thus making the cells more susceptible to Ca^2+^-induced caspase 3 activation and cell death [76].

Alterations in genes related to mitochondrial energy metabolism and apoptosis were reported in young transgenic AD mice, which persisted throughout adulthood [77]. Levels of proteins regulating mitochondrial biogenesis, such as peroxisome proliferator-activated receptor (PPAR)-γ coactivator-1α (PGC-1α), nuclear respiratory factors 1 (NRF1) and 2 (NRF2), and mitochondrial transcription factor A (Tfam), were significantly reduced in human AD hippocampus and cellular models overexpressing APP Swedish mutation [78,79]. Pedros et al. [80] demonstrated early impairment in genes involved in glucose metabolism and mitochondrial function, including AMP-activated protein kinase (AMPK), PGC-1α, NRF1, and NRF2, as well as alterations in oxidative phosphorylation (OXPHOS) complexes in the pre-plaque APP/PS1 mice. Thus, mitochondrial function and activity are reduced, and Aβ further entangles mitochondria in various ways in AD. On the other hand, increasing PGC-1α via PPARs or sirtuins reduces Aβ plaques and is neuroprotective in AD, further indicating the importance of maintaining mitochondrial bioenergetics for a healthy neuronal function [81,82,83,84].

## 3. Glucose Metabolism in AD

Previous studies have shown a link between brain glucose metabolism impairments and AD pathogenesis [85,86,87,88]. Arterio-venous difference studies have provided the first quantitative evaluation of reduced glucose metabolism in the AD brain [89,90,91]. However, direct evidence has come from PET imaging studies with [^18^F]-fluoro-deoxyglucose (FDG) demonstrating that AD is associated with global reductions in brain glucose metabolism, relative to normal, healthy control brains [86,92]. These observations have been reinforced by multiple other studies over time [93,94,95,96]. The reduction in glucose utilization in AD brains could be a consequence of reduced glycolysis, neuronal loss, as well as a reduced glucose uptake [6,97]. Concentrations of GLUT1 and GLUT3 are reduced in the brains of AD patients and correlate with diminished brain glucose uptake and subsequent cognitive decline [98,99,100,101,102,103,104]. Aβ interferes with GLUT3 expression and membrane translocation and impairs glucose uptake [105,106]. GLUT3 membrane translocation is regulated by AMPK, which is inhibited by Aβ [107,108,109]. Higher brain glucose concentration, reduced glycolytic flux, and lower GLUT3 levels are related to the severity of AD pathology and the degree of AD symptoms [95]. In mouse models of AD, a reduction of GLUT1 levels worsens amyloid pathology, neurodegeneration, and cognitive function [110]. The insulin-regulated GLUT4 plays a key role in memory acquisition in the hippocampus and in brain insulin resistance, indicating a possibility that impairments in GLUT4 trafficking between the cytosol and plasma membrane in the brain could lead to cognitive impairment [111,112]. Stimulating glucose metabolism in specific brain regions results in cognitive improvements in spatial-reference learning and memory, memory flexibility, and novel object-recognition tests in AD mice [113].

**Figure 2:**
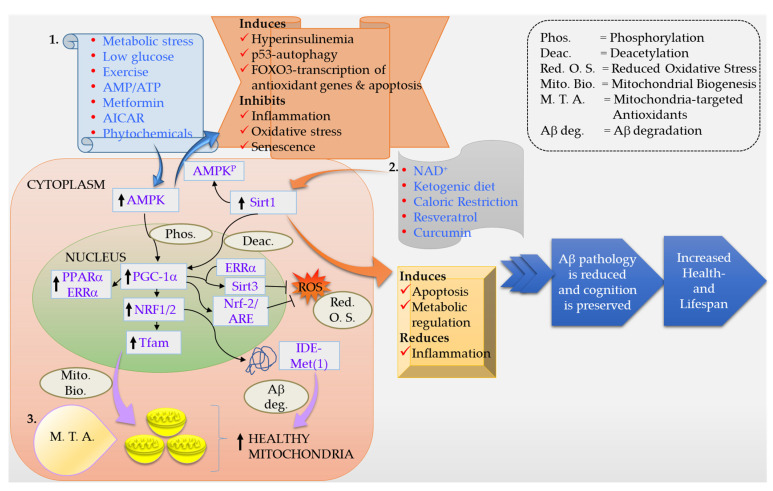
Schematic representation of various factors and processes involved in the mechanism of neuroprotection in AD. Various therapeutic approaches and physiologic and extracellular signals are shown in boxes 1 and 2 that induce the activity of AMPK and Sirt1, respectively. Downstream changes are reflected in expression of PGC-1α, NRF1, NRF2, Tfam, Sirt3, etc. IDE-Met(1) metabolizes mitochondrial Aβ and is regulated by the PGC-1α-NRF1 pathway. As a result of the activation of the AMPK-PGC-1α-NRF1-Sirtuins cascade, mitochondrial biogenesis, autophagy, Aβ degradation, and metabolic regulation are induced, and oxidative stress is reduced. A direct delivery of therapeutic molecules is now achievable, owing to the nano-carriers and mitochondria-targeted antioxidants. All of these approaches have shown success in various clinical and preclinical studies and hold promise for the future.

Furthermore, in early or intermediate stages of AD, brain and CSF levels of insulin are also decreased [114,115,116]. Thus, in the early stages, AD is marked by deficits in cerebral glucose utilization and energy metabolism, and as AD progresses, impairments in insulin signaling, insulin-responsive gene expression, glucose utilization, and metabolism worsen [85,117,118,119,120,121,122,123,124]. Mitochondrial Aβ is metabolized by the long isoform of the insulin-degrading enzyme (IDE), called IDE-Met(1) [125,126]. IDE-Met(1) is present in the brain, and its expression is regulated by the PGC-1α-NRF1 pathway (Figure 2). Studies of postmortem brains showed a strong positive correlation between PGC-1α-NRF1 and long IDE isoform transcripts in non-demented brains and a weaker correlation in AD, suggesting an impairment of this route [87]. In vitro inhibition of IDE increased mitochondrial Aβ and impaired mitochondrial respiration, alterations that were restored by blocking mitochondrial Aβ production or inducing mitochondrial biogenesis. These results showed that mitochondrial biogenesis regulates mitochondrial Aβ production [87]. Investigations of human postmortem brains revealed significant AD stage-dependent declines in insulin and insulin-like growth factors, type 1 (IGF-1) polypeptides (growth factors) and receptors [119,127,128,129,130]. As human AD-associated abnormalities in insulin and IGF-1 signaling are highly reminiscent of type 1 and type 2 diabetes mellitus, though they selectively involve the brain, de la Monte and colleagues have called AD ‘type 3 diabetes’ [85,131,132,133].

## 4. Oxidative Damage in AD

Another cardinal feature of AD pathogenesis is the extent and accumulation of oxidative damage that takes place alongside alterations in glucose metabolism and mitochondrial dysfunction in the brains of transgenic animal models and patients with AD [134,135,136,137,138,139,140,141]. Increased free radicals and carbonylated proteins were observed in concert with increased mitochondrial dysfunction in young transgenic AD mice [38]. Oxidative stress is consistently observed in AD [141,142,143,144,145]. Increased lipid peroxidation precedes amyloid plaque formation in an animal model of AD and is a recurrent feature of preclinical AD [146,147,148,149]. In rat cultured neurons, Aβ induced conjugation of 4-hydroxynonenal (HNE, an aldehyde product of lipid peroxidation) to GLUT3, thereby inhibiting its function and leading to ATP depletion [150]. Thus, defective glucose metabolism, mitochondrial dysfunction, and oxidative stress are upstream alterations that occur prior to the onset of any discernible neuropathology in AD. It is entirely possible that chronic reductions in glucose uptake and metabolism trigger mitochondrial dysfunction, which, in turn, results in oxidative stress. Oxidative stress further results in inhibition of glycolysis and produces mitochondrial dysfunction. On the other hand, mitochondrial biogenesis regulates mitochondrial Aβ production, suggesting mitochondrial dysfunction to be an upstream event in inducing AD pathology [151,152]. In either scenario, supplementing and supporting mitochondrial function, boosting mitochondrial bioenergetics, and suppression of oxidative stress promise to be attractive therapeutic avenues, which are discussed further below. Later on, we discuss lifestyle and diet changes that help in re-energizing and rejuvenating mitochondrial functions, maintaining overall health and well-being, and thus helping to combat AD.

## 5. Mitochondria-Targeted Therapies in AD

Mitochondria are the source, as well as the sink, for ROS. Any disruption in the finely tuned balance between ROS production and scavenging results in damage to macromolecules and disrupts signaling within the cell [153]. Thus, a logical line of therapy has been the use of antioxidants in prevention or therapy for AD [154,155,156,157,158]. However, the use of antioxidants has given conflicting results, which has been ascribed to the inability of these compounds to cross the blood-brain barrier (BBB) and a failure to reach the desired therapeutic concentrations at the site of ROS production, i.e., mitochondria [159,160,161,162,163,164,165,166,167]. An obvious answer to this problem has been to use compounds/molecules that readily traverse the BBB and selectively target mitochondria to reduce ROS production and thereby reduce oxidative damage (Figure 2) [168]. Recently, Perez Ortiz and Swerdlow presented a very informative summary of data on AD clinical trials with therapeutic interventions targeting mitochondria [11]. Mitochondria-targeted antioxidants have been developed and used in AD—for example, the triphenylphosphonium-based antioxidants (MitoQ, MitoVitE and MitoPBN), the cell-permeable, small peptide-based antioxidant SS-31 and other SS-tetra peptides, MitoPeroxidase (MitoEbselen), and choline esters of glutathione and N-acetyl-L-cysteine [169,170,171,172,173,174,175]. A few of these have shown beneficial antioxidant, as well as neuroprotective effects, summarized below.

### 5.1. MitoQ

MitoQ [mitoquinone mesylate: (10-(4,5-dimethoxy-2-methyl-3,6-dioxo-1,4-cyclohexadienlyl) decyl triphenylphosphonium methanesulfonate)] is a ubiquinone derivative targeted to mitochondria by covalent attachment to a lipophilic triphenylphosphonium cation through an aliphatic carbon chain. MitoQ concentrates several hundredfold in the mitochondrial matrix due to the large mitochondrial membrane potential where the ubiquinone moiety gets inserted into the lipid bilayer and is reduced by the respiratory chain to ubiquinol [173,176,177,178]. After detoxifying a ROS, the ubiquinol moiety is regenerated by the respiratory chain, enabling its antioxidant activity to be recycled. MitoQ improved cognition, reduced oxidative stress, increased synaptic markers, reduced gliosis, and reduced levels of amyloid and early neuropathology in a transgenic mouse model of AD [179]. In older AD mice, mitoQ treatment improved memory retention, prevented synaptic loss, reduced oxidative stress, reduced astro- and micro-gliosis, and reduced tau and Aβ accumulation, caspase activation, and tau hyperphosphorylation [180]. MitoQ also increased the lifespan of AD mice to a similar extent as control mice [180]. In primary neurons of APP transgenic mice and in neuronal cell lines treated with MitoQ and then exposed to Aβ, abnormal expression of peroxiredoxins and mitochondrial structural genes was prevented, and mitochondrial function and neurite outgrowth were normal. These findings suggest that MitoQ protected neurons from Aβ toxicity [181]. MitoQ also extends lifespan, delays Aβ-induced paralysis, ameliorates depletion of the mitochondrial lipid cardiolipin, protects complexes I and IV of the ETC, and has protective effects on life- and healthspan of a transgenic *Caenorhabditis elegans* model of AD [182]. MitoQ has been assessed in clinical trials, including a trial of 128 Parkinson’s disease (PD) patients over a 12-month period, as well as in a few other disease modalities, and does appear to be tolerable [183]. One pilot study of MitoQ in Alzheimer’s patients is underway and currently recruiting (https://clinicaltrials.gov/ct2/show/study/NCT03514875?term=mitoq (accessed on 24 October 2021)).

### 5.2. SS-31

SS-31 (also known as Elamipretide^®^, Bendavia^®^ and MTP-131) is one of the cell-permeable tetra-peptides out of a series of SS (named after the inventors Szeto-Schiller)] peptides—SS-02, SS-19, SS-20, SS-31 [184,185]. SS-31 inhibited lipid peroxidation, scavenged H_2_O_2_ in vitro, protected neurons from Ca^2+^-induced mitochondrial depolarization and swelling, decreased the release of cytochrome c in isolated organelles, and protected against ischemia reperfusion injury in the guinea pig heart [185]. Pretreatment of neuronal cell lines and primary neurons from AD-transgenic mice exposed to Aβ with SS-31 resulted in the partial rescue of various mitochondrial dysfunction and oxidative stress parameters [181]. SS-31 reversed the defects in anterograde trafficking of mitochondria and the excess mitochondrial fission that occurred in Aβ-exposed neurons [181]. Reddy et al. [186] further showed that SS-31 administered to APP mice via intraperitoneal injections crossed the BBB and reached mitochondrial sites of free radical production. SS-31 reduced Aβ production and mitochondrial dysfunction, restored mitochondrial dynamics, and enhanced mitochondrial biogenesis and synaptic activity in AD mice [186,187]. SS-31 was recently shown to ameliorate mitochondrial dysfunction and synaptic and memory impairment induced by neuroinflammation [188,189]. Despite its success in preclinical trials in aging and related health conditions, clinical trials of SS-31 have not been successful so far (tested in heart failure and primary mitochondrial myopathy).

### 5.3. SkQ

SkQ is a lipophilic cation, linked via saturated hydrocarbon chain to an antioxidant, i.e., a mitochondrially targeted antioxidant. Similar to SS-31, it is named after its inventor, Vladimir Skulachev. SkQ has a plastoquinone moiety that is a stronger antioxidant than ubiquinone. Due to its lipophilic properties, SkQ can effectively penetrate through various cell membranes and accumulate into the negatively charged mitochondrial matrix. SkQ is able to protect cells from death due to oxidative stress and is effective as a treatment of age-related diseases in animals [190,191]. SkQ1 improved healthspan and lifespan in mitochondrial DNA (mtDNA) mutator mice [192]. Neuroprotective effects of SkQ1 were demonstrated in the senescence-accelerated OXYS rat, a model of aging featuring an overproduction of free radicals, lipid peroxidation, protein oxidation, DNA damage, and a variety of neurodegenerative features [193,194,195,196]. SkQ1 treatment in OXYS rats preserved hippocampal neuronal integrity, improved mitochondrial parameters, including increased enzymatic activity of ETC complexes I and IV, and reduced mitochondrial swelling and lipofuscin accumulation in the hippocampal CA1 neurons [197]. Interestingly, SkQ1 reduced Aβ levels and tau hyperphosphorylation, promoted neurogenesis and cell survival, prevented synaptic pathology, and improved cognitive function in vivo [197,198,199,200,201]. Other derivatives and mixtures of SkQ also show neuroprotective effects in Aβ-induced decay of long-term potentiation in rat hippocampal slices and in an open focal trauma-induced neurological deficit in rats [202,203]. SkQ1 is currently being tested in a phase II trial by Mitotech S.A. for dry eye disease and is under study for multiple sclerosis.

### 5.4. MitoVitE

MitoVitE (also called TPPB) consists of alpha-tocopherol linked to the triphenylphosphonium (TPP) cation by a hydrocarbon chain, enabling its rapid uptake through the plasma and mitochondrial membranes and accumulation within mitochondria, as a result of the large membrane potential (negative inside) across the IMM. MitoVitE accumulates in all major organs of mice and rats after oral, intraperitoneal, or intravenous administration [204,205]. MitoVitE is effective in reducing mitochondrial damage when induced by conditions involving oxidative stress, such as rat models of sepsis, and in neuropathy or pain [201,206,207,208,209,210,211]. Specifically, MitoVitE protected against loss of mitochondrial membrane potential, reduced metabolic activity, and loss of glutathione in rat dorsal root ganglion cells in vitro [208].

### 5.5. MitoTEMPO

MitoTEMPO is composed of the antioxidant piperidine nitroxide TEMPO, linked to the lipophilic cation triphenylphosphonium (TPP), giving MitoTEMPO the ability to pass through lipid bilayers with ease and accumulate several hundredfold in mitochondria [212]. TEMPO is a superoxide dismutase (SOD) mimetic, while TPP is a membrane-permeant cation. The mitochondria-targeted antioxidant was tested against the toxicity of Aβ in primary cultured neurons. MitoTEMPO resolved the Aβ-induced mitochondrial oxidative stress and ameliorated mitochondrial dysfunction [213]. In another study, MitoTEMPO relieved neuropathic pain by protecting mitochondria against oxidative stress, significantly increased expression of mitochondrial fusion proteins (mitofusin 1 (Mfn1) and optic atrophy 1 (OPA1)), and significantly decreased expression of fission markers (dynamin related protein 1 (Drp1) and fission 1 (Fis1)) [214]. MitoTEMPO prevented oxalate-induced injury by inhibiting mitochondrial dysfunction and decreasing oxidative stress in NRK-52E cells. Additionally, the inhibition of mitochondrial ROS with MitoTEMPO reduced diabetic cardiomyopathy [215,216]. Aβ and oxidative stress both induce activation of the p38 MAP kinase, and its phosphorylation links neuronal and synaptic perturbation [217,218,219,220]. MitoTEMPO inhibited phosphorylation of the p38 MAP kinase, suppressed ROS production, and increased COX activity and ATP levels in Aβ-treated hippocampal slices from mice transgenic for Endophilin A1 (a protein enriched in synaptic terminals that increases Aβ) [221]. MitoTEMPO has neuroprotective effects against glutamate cytotoxicity through its direct free radical-scavenging activity and suppresses autophagic flux via the phosphatidylinositol-3-kinase (PI3K)/protein kinase B (Akt) and the mammalian target of rapamycin (mTOR) (PI3K/Akt/mTOR) signaling pathway in neuroblastoma SH-SY5Y cells [222].

### 5.6. MitoApocynin (MitoApo)

A novel mitochondrially targeted antioxidant and NADPH oxidase (NOX) inhibitor, MitoApo, is neuroprotective in a selective knockout of Tfam in dopaminergic neurons, the MitoPark mice, and cell culture models of neuroinflammation and mitochondrial dysfunction [223]. Oral administration of MitoApo showed excellent central nervous system bioavailability and significantly improved locomotor activity and coordination in MitoPark mice. Importantly, MitoApo partially attenuated severe nigrostriatal degeneration, improved mitochondrial function, and inhibited NOX2 activation, oxidative damage, and neuroinflammation in MitoPark mice [223]. MitoApo was effective in this and other models of PD, as well as in a kainic acid-induced model of excitotoxicity [224,225,226]. Biodegradable polyanhydride-based nano-carriers can provide sustained delivery of diverse payloads to organelles with reduced toxicity and increased bioavailability [227]. Biodegradable nanomaterials have been extensively evaluated for drug delivery across the BBB [228,229]. Taking advantage of the ability of nano-carriers to cross highly selective biological barriers with intracellular targeting, Brenza et al. [230] demonstrated that polyanhydride nanoparticles can efficiently deliver MitoApo to a mesencephalic neuronal cell line and to primary cortical neurons, where it effectively protects against oxidative stress-induced neuronal damage. A recent study, however, showed potential toxic effects of MitoApo, which could be a dealbreaker [231].

### 5.7. Mdivi-1 (Mitochondrial Division Inhibitor-1)

Mitochondrial fission and fusion balance are tipped in favor of fission in AD, leading to excessive mitochondrial fragmentation and dysfunctional mitochondria [27,39,232]. Drp1 colocalizes with Aβ and interacts with Aβ monomers and oligomers in AD patients, and these abnormal interactions increase with disease progression [233,234]. Levels of mitochondrial fission proteins Drp1 and Fis1 are increased in the hippocampi of APP mice, while mitochondrial fusion proteins Mfn1, Mfn2, and Opa1 are significantly decreased and Aβ directly interferes with the transcription and expression of these genes [57,235]. Mdivi-1 is a derivative of quinazolinone, namely 3-(2,4-dichloro-5-methoxyphenyl)-2-thioxoquinazoline-4-one, and a cell-permeable selective inhibitor of Drp1 GTPase activity that blocks the self-assembly and polymerization of Drp1, resulting in a reversible formation of elongated and tubular mitochondria [236,237]. The inhibition of Drp1 by mdivi-1 prevented Aβ-mediated mitochondrial dysfunction and synaptic depression in neurons and significantly reduced Aβ deposition, lipid peroxidation, and BACE1 (β-secretase enzyme crucial for Aβ production) expression in the brain of AD mice [238]. Furthermore, mdivi-1 alleviates mitochondrial fragmentation, loss of mitochondrial membrane potential, ROS production, and ATP reduction in Aβ-treated neurons and prevents neuropathology and cognitive decline in APP/PS1 mice [238]. In another parallel study of AD-transgenic mice, mdivi-1 treatment was shown to rescue both mitochondrial fragmentation and distribution deficits and improve mitochondrial function in CRND8 neurons both in vitro and in vivo [239]. The amelioration of mitochondrial dynamics deficits by mdivi-1 treatment markedly decreased extracellular amyloid deposition, prevented the development of cognitive deficits in the Y-maze test, and improved synaptic parameters [239]. Reddy et al. [240] showed that pretreatment of N2a cells with mdivi-1 had a more protective effect than the post-Aβ-challenged mdivi-1 treatment with respect to reduced mitochondrial dysfunction, maintenance of cell viability, mitochondrial dynamics, mitochondrial biogenesis, and synaptic activity. Moreover, a combined treatment of mitochondria-targeted antioxidant SS-31 and mdivi-1 was more effective than either treatment alone in AD neurons [241]. Neuroprotective effects of mdivi-1 were also shown in a rat model of PD [242]. The observations of inhibition of Drp1 by mdivi-1 were, however, challenged by a study showing that mdivi-1 reversibly inhibits mitochondrial complex I-dependent oxygen consumption instead of acting as a specific Drp1 GTPase inhibitor. Mdivi-1 additionally decreases ETC complex-I-dependent ROS production, which is presumed to have resulted in the neuroprotective effects observed following mdivi-1 treatment in various models [243].

### 5.8. Ceramide and Mitochondrial Fission

Ceramides are lipids composed of sphingosine and a fatty acid, varying in length from C14 to C26, that act as second messengers in regulating several biochemical events, including terminal differentiation, proliferation of neurons, and cellular aging and death [244,245]. Ceramide is elevated in the brains of patients with AD [246,247,248,249]. A cell-permeable analog of ceramide, C6-ceramide, was shown to increase the generation of Aβ by post-translationally stabilizing BACE1 [250]. Ceramide transfer proteins bind to APP and reduce Aβ aggregation and neurotoxicity in vitro and in vivo [251]. Astrocyte-derived extracellular vesicles in AD mice and AD patients were shown to be enriched in ceramide and associated with Aβ [252]. These vesicles were further shown to be transported to mitochondria, where they induced Drp1, mediated binding of Aβ to VDAC, and activated caspases. Thus, ceramide enrichment enhanced Aβ interaction with the astrocyte-derived extracellular vesicles in AD, which, in turn, resulted in neurite fragmentation and neuronal cell death [252]. These studies indicate an important role for ceramide in inducing Aβ-mediated toxicity across various AD models and in patients with AD. Recently, the synthetic sphingolipid SH-BC-893 was shown to be a rapid and effective inhibitor of ceramide-induced mitochondrial fission, which works by potentially influencing the recruitment of Drp1 to the OMM ([253], commentary in: [254]). Treatment with SH-BC-893 preserved mitochondrial function in ceramide-treated cells and protected against ER stress induced by ceramide-mediated mitochondrial fission. Additionally, SH-BC-893 mimics the effects of caloric restriction by reducing food intake and thereby producing weight loss [253]. Although the above-mentioned beneficial effects were seen in diet-induced obesity, SH-BC-893 seems to be of great potential and needs to be tested urgently for the treatment of AD.

### 5.9. Other Inhibitors of Mitochondrial Fission

Other small-molecule inhibitors with more potent Drp1-inhibitory effects were identified by chemical library screening and structural optimization [255,256]. Numadate et al. [257] reported 3-[2,6-diethylphenyl]quinazoline-2,4-dione (*PAQ-22*). Mallat et al. [258] identified a novel class of 1H-pyrrole-2-carboxamide compounds that directly inhibit assembly-stimulated Drp1 GTPase activity in vitro. Based on the molecular docking study of the Aβ and Drp1 protein complex, Kuruva et al. [259] designed a novel Drp1 inhibitor named *DDQ* (diethyl (3,4-dihydroxyphenethylamino) quinolin-4-yl]methylphosphonate). DDQ inhibited the Aβ and Drp1 interaction, reduced cellular levels of Aβ oligomers, and improved mitochondrial function and cell viability in cell-based models of AD [259]. Gan et al. [219] used AD cybrid cells (cytoplasmic hybrid (cybrid) neurons with incorporated platelet mitochondria from AD and age-matched non-AD human subjects into mtDNA-depleted neuronal cells) to study changes in mitochondrial morphology and function. They demonstrated that blockade of the mitochondrial fission dynamin-like protein 1 (DLP1, which is another name for Drp1), by a genetic manipulation with a dominant negative DLP1 (*DLP1*(*K38A*)), its knock-down with *siRNA-DLP1*, or inhibition of mitochondrial division with mdivi-1 attenuated mitochondrial functional defects observed in AD cybrid cells [219]. Yet another selective inhibitor of GTPase activity of dynamin1, dynamin2, and Drp1 was identified upon screening of about 16,000 small molecules and named *dynasore* [210]. These compounds have neurotherapeutic potential if they are readily permeable through the BBB and could be investigated and studied further.

## 6. Lifestyle Modifications

From the preceding discussion, it is clear that sporadic AD is of complex etiology: defective glucose metabolism, mitochondrial dysfunction and oxidative stress all seem to contribute to its neuropathology. Even in the early onset AD associated with a known genetic cause, this trifecta of events appears well before the onset of neuropathological abnormalities such as Aβ deposition and cognitive dysfunction. Lifestyle interventions, specifically diet and exercise, not only act at the mitochondrial level but also help in regulating glucose metabolism and in maintaining a healthy weight and healthy mind and are therefore likely the most efficacious interventions to treat AD. Dietary restriction and exercise enhance synaptic plasticity, neurogenesis, and cognitive performance and reduce oxidative stress in mice [260,261,262,263].

### 6.1. Exercise

Exercise affects redox regulation, enhancing endogenous antioxidant capacities in the brain of rats [264,265]. Long-term treadmill exercise improved cognitive deficits in the APP/PS1 transgenic mouse model of AD, paralleled by enhanced long-term potentiation (LTP) [266]. Furthermore, five months of treadmill exercise resulted in a robust reduction in Aβ deposition and tau phosphorylation, accompanied by a significant decrease in APP phosphorylation and PS1 expression in the hippocampus of APP/PS1 mice [267,268]. Regular aerobic exercise improves executive function and attention processing and increases cortical thickness, speed memory, and learning in young, as well as older, adults [269,270,271,272,273,274,275,276]. Physical exercise is associated with enhanced volume of the prefrontal and medial temporal cortices, as well as the hippocampus, in elderly people [277,278,279,280]. Regular physical exercise prevents memory and cognitive decline in affected patients, exerts anti-inflammatory effects, improves the brain redox status, and ameliorates cardiovascular risk factors (e.g., reduced vascular flow, diabetes) involved in the pathogenesis of AD (Reviewed in: [281,282]). Exercise also promotes neurogenesis via increases in exercise-induced metabolic factors (e.g., ketone bodies, lactate) and muscle-derived myokines (cathepsin-B, irisin), which, in turn, stimulate the production of neurotrophins such as brain-derived neurotrophic factor [281]. A multimodal physical exercise program reduced fall risk and produced an improvement in gait, balance, and bone mineral density in the short and medium term in institutionalized patients with AD [283]. Regular physical exercise protects against AD by inhibiting different pathophysiological molecular pathways implicated in AD [284]. The beneficial effects of exercise in reducing the levels of Aβ were reviewed recently [285].

Regular endurance exercise improves mitochondrial health, mitochondrial plasticity, and mitochondrial biogenesis and respiration [286,287]. It also enhances antioxidant capacities and the affinity of mitochondria for oxygen, improving healthy aging ([288,289,290,291] and references therein). Moderate-to-high-intensity exercise reduced neuropsychiatric symptoms in patients with mild AD and preserved cognition in a subgroup of patients exercising with high attendance and intensity [292]. In a single-blinded, multi-center randomized controlled trial (ADEX), the intervention group received supervised moderate-to-high-intensity aerobic exercise 1 h × 3/week for 16 weeks. Aerobic exercise improved cardiorespiratory fitness, single-task physical performance, dual-task performance, and exercise self-efficacy in patients with mild AD [293,294]. Another study of the effects of exercise showed improved memory performance and reduced hippocampal atrophy, along with improved cardiorespiratory fitness, in AD patients [295]. Moderate-intensity cycling may reduce the decline in global cognition in older adults with mild-to-moderate AD dementia [296]. A meta-analysis study using a random-effects model compared different quantities of physical activity and exercise interventions for AD in detail and concluded that physical activity and exercise can improve cognition in older adults with AD [297].

In patients with AD, physical training significantly improved the judgment and problem-solving domains of the memory score [298]. While general mental health, memory, orientation, and home/hobby domains were improved slightly, the neurotrophin levels remained unaltered. Significantly, the markers of protein integrity, as well as nitrite levels and interleukin-4 levels, increased, while catalase activity and ROS levels decreased following physical training in patients with AD. The levels of neuron-specific enolase, a marker of neuronal damage, decreased following exercise training in these patients [298]. Studies examining the effectiveness of aerobic exercise as a cognitive intervention for older adults with MCI reached a similar conclusion that participation in regular aerobic exercise/dance can improve cognitive function in older adults with MCI [299,300,301,302]. In short, physical exercise training could be a safe, efficacious, and economic approach to manage AD. Standardized protocols, larger and more rigorous, randomized controlled trials with longer-term followups may provide better insight into the effects of aerobic exercise on cognitive deterioration in people with AD and MCI. Methods of assessment of the serum biomarkers associated with the redox status, neurotrophin levels, and inflammatory system, etc., should be uniform.

### 6.2. Diet

The Finnish Geriatric Intervention Study to Prevent Cognitive Impairment and Disability (FINGER) showed that a multidomain intervention, including diet, exercise, cognitive training, and vascular risk monitoring, could improve or maintain cognitive functioning in at-risk elderly people [303,304]. Dietary restriction in animals extends their lifespan and increases the resistance of neurons to degeneration [305]. Scarmeas et al. [306] examined the association between the Mediterranean diet (mainly composed of fruits, vegetables, legumes, a moderate amount of ethanol and dairy products, and omega-3 fatty acids) and AD using data from the Washington Heights-Inwood Columbia Aging Project (WHICAP). A higher adherence to the Mediterranean diet was associated with a reduced risk for developing mild cognitive impairment (MCI) and AD and a reduced risk of progression from MCI to AD [307]. Higher adherence to the Mediterranean diet was found to be associated with higher global cognitive performance and brain structural integrity as well as decreased risk of AD and vascular dementia in older adults. However, findings in a Dutch cohort were contrary to this [308,309,310,311]. Further prospective cohort studies with longer followup and randomized controlled trials are warranted.

High dietary intake of long-chain polyunsaturated fatty acids (PUFAs), specifically docosahexaenoic acid (DHA) and eicosapentaenoic acid (EPA), is associated with lower risk of AD [312]. Dietary fish or fish oil rich in omega-3 fatty acids, DHA, and EPA affect psychiatric and behavioral symptoms in AD [313,314]. Administration of omega-3 fatty acids to patients with mild to moderate AD did not delay the rate of cognitive decline, although positive effects were observed in a small group of patients with very mild AD and in MCI [315,316]. Dietary intake of various parts of plants, including leaves, fruits, bark, and roots have long been used in Indian Ayurvedic medicine to enhance memory and cognition, as well as to treat various medical conditions. These preparations have also been tried in various formulations in AD and showed promise—for example, mulberry leaf extract, *Ginkgo biloba* extract, green and black teas, fruit polyphenols such as blueberry or pomegranate juice [317,318,319,320]. Seven helpful guidelines related to a healthy diet and exercise habits were compiled from the speakers’ presentations at the International Conference on Nutrition and the Brain, Washington, DC, 19–20 July 2013 [321].

Hippocampal pyramidal neurons from mice, inducibly expressing a mutated form of the mtDNA-repair enzyme UNG1, showed improved markers of mitochondrial biogenesis, dynamics, and function upon being fed a ketogenic diet (KD; high-fat, low-carbohydrate). These improvements were due to an upregulation of PGC-1α, sirtuin 3 (Sirt3), and uncoupling protein 2 (UCP2) [322]. Similarly, cultured rat hippocampal neurons and human fibroblasts with H_2_O_2_ induced oxidative stress when exposed to the ketone body β-hydroxybutyrate and showed an increased oxygen consumption rate (OCR) and NAD^+^/NADH ratio. The KD’s high-fat, low-carbohydrate composition reduces glucose utilization and promotes the production of ketone bodies. Ketone bodies are a more efficient energy source than glucose and improve mitochondrial function and biogenesis. One of the benefits of a ketogenic diet is that it increases mitochondrial biogenesis and bioenergetics via the PGC-1α-Sirt3-UCP2 axis [322]. The neuroprotective effects of the ketogenic diet were ascribed to increased neuronal levels of ketone bodies inducing hypoxia-inducible factor-1α (HIF-1α) and sirtuin 1 (Sirt1), in part by increasing cytoplasmic and nuclear levels of Sirt1’s obligate cofactor, NAD^+^ [323,324]. Reduced activity of mTOR was observed in the hippocampi of rats fed a ketogenic diet, an effect plausibly attributable to Sirt1 activation [325]. Increased activities of HIF-1α and Sirt1 and a decrease in mTOR activity could be expected to collaborate in the induction of neuronal macroautophagy, which is beneficial in getting rid of damaged mitochondria. Kashiwaya et al. [326] showed that a transgenic mouse model of AD fed a ketone ester diet exhibited less anxiety and improved cognition, along with reduced amyloid and tau pathologies. On the flip side, mice fed a high-fat, high-cholesterol diet show an increased transcription of β-secretase/BACE1, the rate-limiting enzyme for Aβ generation, which, in turn, is reciprocally regulated by PGC-1α [81,327]. At the same time, fasting reverses these effects by stimulating the Sirt1-PPARγ-PGC-1α and thus suppressing BACE1 transcription and Aβ production [328].

A recent study has shown that decanoic (capric) acid, a key component of the medium-chain triglyceride (MCT) ketosis-inducing diet, decreases activity of the mTOR complex in the absence of insulin and under high-glucose conditions in *ex vivo* rat hippocampi and in tuberous sclerosis complex patient-derived astrocytes [329]. In mild to moderate AD patients, it was shown that the brain can utilize additional ketones as fuel when they are derived from an MCT supplement [330]. These patients consumed a mixture of caprylic acid (octanoic acid) and capric acid, followed by tricaprylin (an octanoate triester of glycerol). Brain ketone ([^11^C]-acetoacetate) and glucose (FDG) uptake were quantified by PET before and after each MCT intervention. In these patients, brain ketone consumption doubled on both types of MCT supplement. Both types of MCT increased total brain energy metabolism by increasing ketone supply without affecting brain glucose utilization. Thus, it was concluded that ketones from MCT compensate for the brain glucose deficit in AD in direct proportion to the level of plasma ketones achieved [330]. Additional studies using quantitative kinetic PET and MRI imaging demonstrated that the deterioration in brain energy metabolism is specific to glucose in MCI and AD, and therefore, a ketogenic diet, along with other interventions, should be helpful in delaying or preventing cognitive decline [331].

Dietary interventions such as caloric restriction (CR) and intermittent fasting are known to prolong life and healthspan in model organisms. CR consists of reducing the daily calorie intake, while maintaining essential nutrients for health, without malnutrition. A CR dietary regimen prevented Aβ peptide production and plaque deposition in multiple models of AD, leading to the reduction of neuronal loss in the hippocampus and the improvement of cognitive deficits [332,333,334,335,336,337,338]. Mechanisms triggered by CR include promotion of anti-amyloidogenic alpha-secretase activity and induction of the NAD^+^-dependent Sirt1 deacetylase and autophagy [332,334,339,340]. Both animal and human studies have shown that CR benefits general health, improves memory and cognition, and slows down the aging process. CR mimetics, as the name indicates, are compounds that mimic the biochemical and functional effects of CR without the need to reduce energy intake [341,342]. Examples of CR mimetics include caffeine, curcumin, dapsone, metformin, rapamycin, resveratrol, and spermidine. Many of these compounds are beneficial in AD. Late-onset, short-term intermittent fasting dietary restriction improved motor coordination and cognitive ability of aging male rats. These changes positively correlated with the decline in the oxidative molecular damage to proteins and enhanced mitochondrial complex IV activity in different regions of the aging brain, as well as peripheral organs [343]. A randomized controlled trial study showed that alternate-day fasting [ADF] improves markers of general health in middle-aged people [344]. Interestingly, ADF increased β-hydroxybutyrate, even on non-fasting days. On fasting days, the pro-aging amino-acid methionine, among others, was periodically depleted, while PUFAs were elevated. These results support further investigation of the effects of ADF in AD and MCI [344]. Finally, a recent study showed that intermittent fasting enhances long-term memory consolidation, adult hippocampal neurogenesis, and expression of longevity gene Klotho [345]. A combination of CR or CR mimetics with diet and exercise could be the key to the puzzle of neuroprotection and regeneration (Figure 2).

## 7. Conclusions and Future Perspectives

AD is the leading cause of dementia in older adults. Currently, no disease-modifying therapies are available, and treatments are limited to symptomatic management. Mitochondrial dysfunction, oxidative stress, and metabolic abnormalities are implicated in the disease pathogenesis. Mitochondria, as the powerhouses of the cell and the guardians of the other cellular pathways crucial for survival, present unique challenges for manipulation. One of the major issues is achieving relevant physiological concentrations of the therapeutic compounds at the site of action, i.e., mitochondria. Scientists have figured out a few ways to do that, either by conjugating the therapeutic molecules to biodegradable nano-carriers or to cell-permeable and mitochondrial membrane-permeable recyclable lipophilic cations [346]. These compounds have met with some success across various cellular and animal models of AD, as well as in patients with MCI and AD, and are being tested further. Lifestyle factors play an important role in the etiopathology of AD. Hypertension, stroke, diabetes, and hypercholesterolemia are increasingly being recognized as risk factors for AD, and AD itself is being described as a metabolic/multifactorial disease [5,6]. There is a shift in AD brains from glucose metabolism to amino acid and fatty acid metabolism [347]. Exercise stimulates bioenergetics and increases fat oxidation in mitochondria. Therefore, lifestyle modifications, including diet and exercise, are another avenue being pursued, with similar success as the mitochondria-targeted compounds. Recent research suggests that restoration of mitochondrial function by physical exercise, an antioxidant diet, or therapeutic approaches can delay the onset and slow the progression of AD [291,348,349,350,351,352,353]. Based on the review of literature presented above, I conclude that a combination of mitochondria-targeted antioxidants with diet and exercise interventions may achieve desirable treatment efficacy and could be the way to proceed in the future. Concerted clinical trials are needed for such therapies.

## References

[1] Selkoe D.J. (2001). Alzheimer’s disease: Genes, proteins, and therapy. Physiol. Rev..

[2] Walsh D.M., Selkoe D.J. (2007). Aβ oligomers—A decade of discovery. J. Neurochem..

[3] Li D.D., Zhang Y.H., Zhang W., Zhao P. (2019). Meta-Analysis of Randomized Controlled Trials on the Efficacy and Safety of Donepezil, Galantamine, Rivastigmine, and Memantine for the Treatment of Alzheimer’s Disease. Front. Neurosci..

[4] Stanga S., Caretto A., Boido M., Vercelli A. (2020). Mitochondrial Dysfunctions: A Red Thread across Neurodegenerative Diseases. Int. J. Mol. Sci..

[5] Iturria-Medina Y., Carbonell F.M., Sotero R.C., Chouinard-Decorte F., Evans A.C., Alzheimer’s Disease Neuroimaging Initiative (2017). Multifactorial causal model of brain (dis)organization and therapeutic intervention: Application to Alzheimer’s disease. NeuroImage.

[6] Veitch D.P., Weiner M.W., Aisen P.S., Beckett L.A., Cairns N.J., Green R.C., Harvey D., Jack C.R., Jagust W., Morris J.C. (2019). Understanding disease progression and improving Alzheimer’s disease clinical trials: Recent highlights from the Alzheimer’s Disease Neuroimaging Initiative. Alzheimers Dement..

[7] Beal M.F. (1998). Mitochondrial dysfunction in neurodegenerative diseases. Biochim. Biophys. Acta.

[8] Offen D., Elkon H., Melamed E. (2000). Apoptosis as a general cell death pathway in neurodegenerative diseases. J. Neural Transm..

[9] Bezprozvanny I., Mattson M.P. (2008). Neuronal calcium mishandling and the pathogenesis of Alzheimer’s disease. Trends Neurosci..

[10] Monzio Compagnoni G., Di Fonzo A., Corti S., Comi G.P., Bresolin N., Masliah E. (2020). The Role of Mitochondria in Neurodegenerative Diseases: The Lesson from Alzheimer’s Disease and Parkinson’s Disease. Mol. Neurobiol..

[11] Perez Ortiz J.M., Swerdlow R.H. (2019). Mitochondrial dysfunction in Alzheimer’s disease: Role in pathogenesis and novel therapeutic opportunities. Br. J. Pharmacol..

[12] Wojsiat J., Prandelli C., Laskowska-Kaszub K., Martin-Requero A., Wojda U. (2015). Oxidative Stress and Aberrant Cell Cycle in Alzheimer’s Disease Lymphocytes: Diagnostic Prospects. J. Alzheimers Dis..

[13] Perry E.K., Perry R.H., Tomlinson B.E., Blessed G., Gibson P.H. (1980). Coenzyme A-acetylating enzymes in Alzheimer’s disease: Possible cholinergic ‘compartment’ of pyruvate dehydrogenase. Neurosci. Lett..

[14] Sorbi S., Bird E.D., Blass J.P. (1983). Decreased pyruvate dehydrogenase complex activity in Huntington and Alzheimer brain. Ann. Neurol..

[15] Gibson G.E., Sheu K.F., Blass J.P., Baker A., Carlson K.C., Harding B., Perrino P. (1988). Reduced activities of thiamine-dependent enzymes in the brains and peripheral tissues of patients with Alzheimer’s disease. Arch. Neurol..

[16] Beck S.J., Guo L., Phensy A., Tian J., Wang L., Tandon N., Gauba E., Lu L., Pascual J.M., Kroener S. (2016). Deregulation of mitochondrial F1FO-ATP synthase via OSCP in Alzheimer’s disease. Nat. Commun..

[17] Parker W.D. (1991). Cytochrome oxidase deficiency in Alzheimer’s disease. Ann. N. Y. Acad. Sci..

[18] Kish S.J., Bergeron C., Rajput A., Dozic S., Mastrogiacomo F., Chang L.J., Wilson J.M., DiStefano L.M., Nobrega J.N. (1992). Brain cytochrome oxidase in Alzheimer’s disease. J. Neurochem..

[19] Cenini G., Voos W. (2019). Mitochondria as Potential Targets in Alzheimer Disease Therapy: An Update. Front. Pharmacol..

[20] Maurer I., Zierz S., Moller H.J. (2000). A selective defect of cytochrome c oxidase is present in brain of Alzheimer disease patients. Neurobiol. Aging.

[21] David D.C., Hauptmann S., Scherping I., Schuessel K., Keil U., Rizzu P., Ravid R., Drose S., Brandt U., Muller W.E. (2005). Proteomic and functional analyses reveal a mitochondrial dysfunction in P301L tau transgenic mice. J. Biol. Chem..

[22] Johri A., Beal M.F. (2012). Mitochondrial dysfunction in neurodegenerative diseases. J. Pharmacol. Exp. Ther..

[23] Iwangoff P., Armbruster R., Enz A., Meier-Ruge W. (1980). Glycolytic enzymes from human autoptic brain cortex: Normal aged and demented cases. Mech. Ageing Dev..

[24] Hirai K., Aliev G., Nunomura A., Fujioka H., Russell R.L., Atwood C.S., Johnson A.B., Kress Y., Vinters H.V., Tabaton M. (2001). Mitochondrial abnormalities in Alzheimer’s disease. J. Neurosci..

[25] Tyumentsev M.A., Stefanova N.A., Kiseleva E.V., Kolosova N.G. (2018). Mitochondria with Morphology Characteristic for Alzheimer’s Disease Patients Are Found in the Brain of OXYS Rats. Biochemistry.

[26] Baloyannis S.J. (2006). Mitochondrial alterations in Alzheimer’s disease. J. Alzheimers Dis..

[27] Wang X., Su B., Siedlak S.L., Moreira P.I., Fujioka H., Wang Y., Casadesus G., Zhu X. (2008). Amyloid-beta overproduction causes abnormal mitochondrial dynamics via differential modulation of mitochondrial fission/fusion proteins. Proc. Natl. Acad. Sci. USA.

[28] Zhang L., Trushin S., Christensen T.A., Bachmeier B.V., Gateno B., Schroeder A., Yao J., Itoh K., Sesaki H., Poon W.W. (2016). Altered brain energetics induces mitochondrial fission arrest in Alzheimer’s Disease. Sci. Rep..

[29] Smith M.A., Hirai K., Hsiao K., Pappolla M.A., Harris P.L., Siedlak S.L., Tabaton M., Perry G. (1998). Amyloid-β deposition in Alzheimer transgenic mice is associated with oxidative stress. J. Neurochem..

[30] Casley C.S., Canevari L., Land J.M., Clark J.B., Sharpe M.A. (2002). Beta-amyloid inhibits integrated mitochondrial respiration and key enzyme activities. J. Neurochem..

[31] Angelova P.R., Abramov A.Y. (2018). Role of mitochondrial ROS in the brain: From physiology to neurodegeneration. FEBS Lett..

[32] Li F., Calingasan N.Y., Yu F., Mauck W.M., Toidze M., Almeida C.G., Takahashi R.H., Carlson G.A., Flint-Beal M., Lin M.T. (2004). Increased plaque burden in brains of APP mutant MnSOD heterozygous knockout mice. J. Neurochem..

[33] Sirk D., Zhu Z., Wadia J.S., Shulyakova N., Phan N., Fong J., Mills L.R. (2007). Chronic exposure to sub-lethal beta-amyloid (Aβ) inhibits the import of nuclear-encoded proteins to mitochondria in differentiated PC12 cells. J. Neurochem..

[34] Hauptmann S., Scherping I., Drose S., Brandt U., Schulz K.L., Jendrach M., Leuner K., Eckert A., Muller W.E. (2009). Mitochondrial dysfunction: An early event in Alzheimer pathology accumulates with age in AD transgenic mice. Neurobiol. Aging.

[35] Rui Y., Tiwari P., Xie Z., Zheng J.Q. (2006). Acute impairment of mitochondrial trafficking by beta-amyloid peptides in hippocampal neurons. J. Neurosci..

[36] Rui Y., Zheng J.Q. (2016). Amyloid beta oligomers elicit mitochondrial transport defects and fragmentation in a time-dependent and pathway-specific manner. Mol. Brain.

[37] Cai Q., Tammineni P. (2017). Mitochondrial Aspects of Synaptic Dysfunction in Alzheimer’s Disease. J. Alzheimers Dis..

[38] Calkins M.J., Reddy P.H. (2011). Amyloid beta impairs mitochondrial anterograde transport and degenerates synapses in Alzheimer’s disease neurons. Biochim. Biophys. Acta.

[39] Wang X., Su B., Lee H.G., Li X., Perry G., Smith M.A., Zhu X. (2009). Impaired balance of mitochondrial fission and fusion in Alzheimer’s disease. J. Neurosci..

[40] Reddy P.H., Reddy T.P., Manczak M., Calkins M.J., Shirendeb U., Mao P. (2011). Dynamin-related protein 1 and mitochondrial fragmentation in neurodegenerative diseases. Brain Res. Rev..

[41] Flannery P.J., Trushina E. (2019). Mitochondrial dynamics and transport in Alzheimer’s disease. Mol. Cell Neurosci..

[42] Gillardon F., Rist W., Kussmaul L., Vogel J., Berg M., Danzer K., Kraut N., Hengerer B. (2007). Proteomic and functional alterations in brain mitochondria from Tg2576 mice occur before amyloid plaque deposition. Proteomics.

[43] Manczak M., Park B.S., Jung Y., Reddy P.H. (2004). Differential expression of oxidative phosphorylation genes in patients with Alzheimer’s disease: Implications for early mitochondrial dysfunction and oxidative damage. Neuromol. Med..

[44] Lunnon K., Keohane A., Pidsley R., Newhouse S., Riddoch-Contreras J., Thubron E.B., Devall M., Soininen H., Kloszewska I., Mecocci P. (2017). Mitochondrial genes are altered in blood early in Alzheimer’s disease. Neurobiol. Aging.

[45] Zhang L., Trushin S., Christensen T.A., Tripathi U., Hong C., Geroux R.E., Howell K.G., Poduslo J.F., Trushina E. (2018). Differential effect of amyloid beta peptides on mitochondrial axonal trafficking depends on their state of aggregation and binding to the plasma membrane. Neurobiol. Dis..

[46] Hoshi M., Takashima A., Murayama M., Yasutake K., Yoshida N., Ishiguro K., Hoshino T., Imahori K. (1997). Nontoxic amyloid beta peptide 1-42 suppresses acetylcholine synthesis. Possible role in cholinergic dysfunction in Alzheimer’s disease. J. Biol. Chem..

[47] Faizi M., Seydi E., Abarghuyi S., Salimi A., Nasoohi S., Pourahmad J. (2016). A Search for Mitochondrial Damage in Alzheimer’s Disease Using Isolated Rat Brain Mitochondria. Iran. J. Pharm. Res..

[48] Takeda K., Uda A., Mitsubori M., Nagashima S., Iwasaki H., Ito N., Shiiba I., Ishido S., Matsuoka M., Inatome R. (2021). Mitochondrial ubiquitin ligase alleviates Alzheimer’s disease pathology via blocking the toxic amyloid-beta oligomer generation. Commun. Biol..

[49] Loo D.T., Copani A., Pike C.J., Whittemore E.R., Walencewicz A.J., Cotman C.W. (1993). Apoptosis is induced by beta-amyloid in cultured central nervous system neurons. Proc. Natl. Acad. Sci. USA.

[50] LaFerla F.M., Tinkle B.T., Bieberich C.J., Haudenschild C.C., Jay G. (1995). The Alzheimer’s Aβ peptide induces neurodegeneration and apoptotic cell death in transgenic mice. Nat. Genet..

[51] Morton H., Kshirsagar S., Orlov E., Bunquin L.E., Sawant N., Boleng L., George M., Basu T., Ramasubramanian B., Pradeepkiran J.A. (2021). Defective mitophagy and synaptic degeneration in Alzheimer’s disease: Focus on aging, mitochondria and synapse. Free Radic. Biol. Med..

[52] Mattson M.P., Partin J., Begley J.G. (1998). Amyloid beta-peptide induces apoptosis-related events in synapses and dendrites. Brain Res..

[53] Giovanni A., Keramaris E., Morris E.J., Hou S.T., O’Hare M., Dyson N., Robertson G.S., Slack R.S., Park D.S. (2000). E2F1 mediates death of B-amyloid-treated cortical neurons in a manner independent of p53 and dependent on Bax and caspase 3. J. Biol. Chem..

[54] Goudarzi S., Hosseini A., Abdollahi M., Haghi-Aminjan H. (2021). Insights into Parkin-Mediated Mitophagy in Alzheimer’s Disease: A Systematic Review. Front. Aging Neurosci..

[55] Kim H.S., Lee J.H., Lee J.P., Kim E.M., Chang K.A., Park C.H., Jeong S.J., Wittendorp M.C., Seo J.H., Choi S.H. (2002). Amyloid beta peptide induces cytochrome C release from isolated mitochondria. NeuroReport.

[56] Argueti-Ostrovsky S., Alfahel L., Kahn J., Israelson A. (2021). All Roads Lead to Rome: Different Molecular Players Converge to Common Toxic Pathways in Neurodegeneration. Cells.

[57] Han X.J., Hu Y.Y., Yang Z.J., Jiang L.P., Shi S.L., Li Y.R., Guo M.Y., Wu H.L., Wan Y.Y. (2017). Amyloid β-42 induces neuronal apoptosis by targeting mitochondria. Mol. Med. Rep..

[58] Parks J.K., Smith T.S., Trimmer P.A., Bennett J.P., Parker W.D. (2001). Neurotoxic Aβ peptides increase oxidative stress in vivo through NMDA-receptor and nitric-oxide-synthase mechanisms, and inhibit complex IV activity and induce a mitochondrial permeability transition in vitro. J. Neurochem..

[59] Jia K., Du H. (2021). Mitochondrial Permeability Transition: A Pore Intertwines Brain Aging and Alzheimer’s Disease. Cells.

[60] Crouch P.J., Blake R., Duce J.A., Ciccotosto G.D., Li Q.X., Barnham K.J., Curtain C.C., Cherny R.A., Cappai R., Dyrks T. (2005). Copper-dependent inhibition of human cytochrome c oxidase by a dimeric conformer of amyloid-beta1-42. J. Neurosci..

[61] Caspersen C., Wang N., Yao J., Sosunov A., Chen X., Lustbader J.W., Xu H.W., Stern D., McKhann G., Yan S.D. (2005). Mitochondrial Aβ: A potential focal point for neuronal metabolic dysfunction in Alzheimer’s disease. FASEB J..

[62] Hu W., Wang Z., Zheng H. (2018). Mitochondrial accumulation of amyloid β (Aβ) peptides requires TOMM22 as a main Aβ receptor in yeast. J. Biol. Chem..

[63] Manczak M., Anekonda T.S., Henson E., Park B.S., Quinn J., Reddy P.H. (2006). Mitochondria are a direct site of Aβ accumulation in Alzheimer’s disease neurons: Implications for free radical generation and oxidative damage in disease progression. Hum. Mol. Genet..

[64] Devi L., Prabhu B.M., Galati D.F., Avadhani N.G., Anandatheerthavarada H.K. (2006). Accumulation of amyloid precursor protein in the mitochondrial import channels of human Alzheimer’s disease brain is associated with mitochondrial dysfunction. J. Neurosci..

[65] Hansson Petersen C.A., Alikhani N., Behbahani H., Wiehager B., Pavlov P.F., Alafuzoff I., Leinonen V., Ito A., Winblad B., Glaser E. (2008). The amyloid beta-peptide is imported into mitochondria via the TOM import machinery and localized to mitochondrial cristae. Proc. Natl. Acad. Sci. USA.

[66] Gonzalez-Garcia M., Fusco G., De Simone A. (2021). Membrane Interactions and Toxicity by Misfolded Protein Oligomers. Front. Cell Dev. Biol..

[67] Area-Gomez E., Del Carmen Lara Castillo M., Tambini M.D., Guardia-Laguarta C., de Groof A.J., Madra M., Ikenouchi J., Umeda M., Bird T.D., Sturley S.L. (2012). Upregulated function of mitochondria-associated ER membranes in Alzheimer disease. EMBO J..

[68] Fernandes T., Resende R., Silva D.F., Marques A.P., Santos A.E., Cardoso S.M., Domingues M.R., Moreira P.I., Pereira C.F. (2021). Structural and Functional Alterations in Mitochondria-Associated Membranes (MAMs) and in Mitochondria Activate Stress Response Mechanisms in an In Vitro Model of Alzheimer’s Disease. Biomedicines.

[69] Volgyi K., Badics K., Sialana F.J., Gulyassy P., Udvari E.B., Kis V., Drahos L., Lubec G., Kekesi K.A., Juhasz G. (2018). Early Presymptomatic Changes in the Proteome of Mitochondria-Associated Membrane in the APP/PS1 Mouse Model of Alzheimer’s Disease. Mol. Neurobiol..

[70] Leal N.S., Dentoni G., Schreiner B., Naia L., Piras A., Graff C., Cattaneo A., Meli G., Hamasaki M., Nilsson P. (2020). Amyloid Beta-Peptide Increases Mitochondria-Endoplasmic Reticulum Contact Altering Mitochondrial Function and Autophagosome Formation in Alzheimer’s Disease-Related Models. Cells.

[71] Kravenska Y., Nieznanska H., Nieznanski K., Lukyanetz E., Szewczyk A., Koprowski P. (2020). The monomers, oligomers, and fibrils of amyloid-beta inhibit the activity of mitoBKCa channels by a membrane-mediated mechanism. Biochim. Biophys. Acta Biomembr..

[72] Vaillant-Beuchot L., Mary A., Pardossi-Piquard R., Bourgeois A., Lauritzen I., Eysert F., Kinoshita P.F., Cazareth J., Badot C., Fragaki K. (2021). Accumulation of amyloid precursor protein C-terminal fragments triggers mitochondrial structure, function, and mitophagy defects in Alzheimer’s disease models and human brains. Acta Neuropathol..

[73] Chang S., ran Ma T., Miranda R.D., Balestra M.E., Mahley R.W., Huang Y. (2005). Lipid- and receptor-binding regions of apolipoprotein E4 fragments act in concert to cause mitochondrial dysfunction and neurotoxicity. Proc. Natl. Acad. Sci. USA.

[74] Rhein V., Song X., Wiesner A., Ittner L.M., Baysang G., Meier F., Ozmen L., Bluethmann H., Drose S., Brandt U. (2009). Amyloid-beta and tau synergistically impair the oxidative phosphorylation system in triple transgenic Alzheimer’s disease mice. Proc. Natl. Acad. Sci. USA.

[75] Liang T., Hang W., Chen J., Wu Y., Wen B., Xu K., Ding B., Chen J. (2021). ApoE4 (Δ272-299) induces mitochondrial-associated membrane formation and mitochondrial impairment by enhancing GRP75-modulated mitochondrial calcium overload in neuron. Cell Biosci..

[76] Britti E., Ros J., Esteras N., Abramov A.Y. (2020). Tau inhibits mitochondrial calcium efflux and makes neurons vulnerable to calcium-induced cell death. Cell Calcium.

[77] Reddy P.H., McWeeney S., Park B.S., Manczak M., Gutala R.V., Partovi D., Jung Y., Yau V., Searles R., Mori M. (2004). Gene expression profiles of transcripts in amyloid precursor protein transgenic mice: Up-regulation of mitochondrial metabolism and apoptotic genes is an early cellular change in Alzheimer’s disease. Hum. Mol. Genet..

[78] Qin W., Haroutunian V., Katsel P., Cardozo C.P., Ho L., Buxbaum J.D., Pasinetti G.M. (2009). PGC-1α expression decreases in the Alzheimer disease brain as a function of dementia. Arch. Neurol..

[79] Sheng B., Wang X., Su B., Lee H.G., Casadesus G., Perry G., Zhu X. (2012). Impaired mitochondrial biogenesis contributes to mitochondrial dysfunction in Alzheimer’s disease. J. Neurochem..

[80] Pedros I., Petrov D., Allgaier M., Sureda F., Barroso E., Beas-Zarate C., Auladell C., Pallas M., Vazquez-Carrera M., Casadesus G. (2014). Early alterations in energy metabolism in the hippocampus of APPswe/PS1dE9 mouse model of Alzheimer’s disease. Biochim. Biophys. Acta.

[81] Katsouri L., Lim Y.M., Blondrath K., Eleftheriadou I., Lombardero L., Birch A.M., Mirzaei N., Irvine E.E., Mazarakis N.D., Sastre M. (2016). PPARγ-coactivator-1α gene transfer reduces neuronal loss and amyloid-β generation by reducing beta-secretase in an Alzheimer’s disease model. Proc. Natl. Acad. Sci. USA.

[82] Wang J., Guo M.N., Liu Z.Z., Ma S.F., Liu W.J., Qian J.J., Zhang W.N. (2021). PGC-1alpha reduces Amyloid-beta deposition in Alzheimer’s disease: Effect of increased VDR expression. Neurosci. Lett..

[83] Jamwal S., Blackburn J.K., Elsworth J.D. (2021). PPARγ/PGC1α signaling as a potential therapeutic target for mitochondrial biogenesis in neurodegenerative disorders. Pharmacol. Ther..

[84] Jesko H., Wencel P., Strosznajder R.P., Strosznajder J.B. (2017). Sirtuins and Their Roles in Brain Aging and Neurodegenerative Disorders. Neurochem. Res..

[85] Steen E., Terry B.M., Rivera E.J., Cannon J.L., Neely T.R., Tavares R., Xu X.J., Wands J.R., de la Monte S.M. (2005). Impaired insulin and insulin-like growth factor expression and signaling mechanisms in Alzheimer’s disease—Is this type 3 diabetes?. J. Alzheimers Dis..

[86] Daulatzai M.A. (2012). Quintessential risk factors: Their role in promoting cognitive dysfunction and Alzheimer’s disease. Neurochem. Res..

[87] Leal M.C., Magnani N., Villordo S., Buslje C.M., Evelson P., Castano E.M., Morelli L. (2013). Transcriptional regulation of insulin-degrading enzyme modulates mitochondrial amyloid β (Aβ) peptide catabolism and functionality. J. Biol. Chem..

[88] Daulatzai M.A. (2017). Cerebral hypoperfusion and glucose hypometabolism: Key pathophysiological modulators promote neurodegeneration, cognitive impairment, and Alzheimer’s disease. J. Neurosci. Res..

[89] Lying-Tunell U., Lindblad B.S., Malmlund H.O., Persson B. (1981). Cerebral blood flow and metabolic rate of oxygen, glucose, lactate, pyruvate, ketone bodies and amino acids. Acta Neurol. Scand..

[90] Hoyer S., Oesterreich K., Wagner O. (1988). Glucose metabolism as the site of the primary abnormality in early-onset dementia of Alzheimer type?. J. Neurol..

[91] Ogawa M., Fukuyama H., Ouchi Y., Yamauchi H., Kimura J. (1996). Altered energy metabolism in Alzheimer’s disease. J. Neurol. Sci..

[92] De Leon M.J., George A.E., Ferris S.H., Rosenbloom S., Christman D.R., Gentes C.I., Reisberg B., Kricheff I.I., Wolf A.P. (1983). Regional correlation of PET and CT in senile dementia of the Alzheimer type. AJNR Am. J. Neuroradiol..

[93] Mullins R., Reiter D., Kapogiannis D. (2018). Magnetic resonance spectroscopy reveals abnormalities of glucose metabolism in the Alzheimer’s brain. Ann. Clin. Transl. Neurol..

[94] Kuehn B.M. (2020). In Alzheimer Research, Glucose Metabolism Moves to Center Stage. JAMA.

[95] An Y., Varma V.R., Varma S., Casanova R., Dammer E., Pletnikova O., Chia C.W., Egan J.M., Ferrucci L., Troncoso J. (2018). Evidence for brain glucose dysregulation in Alzheimer’s disease. Alzheimers Dement..

[96] Marchitelli R., Aiello M., Cachia A., Quarantelli M., Cavaliere C., Postiglione A., Tedeschi G., Montella P., Milan G., Salvatore M. (2018). Simultaneous resting-state FDG-PET/fMRI in Alzheimer Disease: Relationship between glucose metabolism and intrinsic activity. NeuroImage.

[97] Chen P., Shen Z., Wang Q., Zhang B., Zhuang Z., Lin J., Shen Y., Chen Y., Dai Z., Wu R. (2021). Reduced Cerebral Glucose Uptake in an Alzheimer’s Rat Model with Glucose-Weighted Chemical Exchange Saturation Transfer Imaging. Front. Aging Neurosci..

[98] Simpson I.A., Vannucci S.J., Maher F. (1994). Glucose transporters in mammalian brain. Biochem. Soc. Trans..

[99] Harr S.D., Simonian N.A., Hyman B.T. (1995). Functional alterations in Alzheimer’s disease: Decreased glucose transporter 3 immunoreactivity in the perforant pathway terminal zone. J. Neuropathol. Exp. Neurol..

[100] Szablewski L. (2021). Brain Glucose Transporters: Role in Pathogenesis and Potential Targets for the Treatment of Alzheimer’s Disease. Int. J. Mol. Sci..

[101] Mooradian A.D., Chung H.C., Shah G.N. (1997). GLUT-1 expression in the cerebra of patients with Alzheimer’s disease. Neurobiol. Aging.

[102] Landau S.M., Harvey D., Madison C.M., Reiman E.M., Foster N.L., Aisen P.S., Petersen R.C., Shaw L.M., Trojanowski J.Q., Jack C.R. (2010). Comparing predictors of conversion and decline in mild cognitive impairment. Neurology.

[103] Jin N., Qian W., Yin X., Zhang L., Iqbal K., Grundke-Iqbal I., Gong C.X., Liu F. (2013). CREB regulates the expression of neuronal glucose transporter 3: A possible mechanism related to impaired brain glucose uptake in Alzheimer’s disease. Nucleic Acids Res..

[104] Kyrtata N., Emsley H.C.A., Sparasci O., Parkes L.M., Dickie B.R. (2021). A Systematic Review of Glucose Transport Alterations in Alzheimer’s Disease. Front. Neurosci..

[105] Uemura E., Greenlee H.W. (2001). Amyloid beta-peptide inhibits neuronal glucose uptake by preventing exocytosis. Exp. Neurol..

[106] Prapong T., Buss J., Hsu W.H., Heine P., West Greenlee H., Uemura E. (2002). Amyloid beta-peptide decreases neuronal glucose uptake despite causing increase in GLUT3 mRNA transcription and GLUT3 translocation to the plasma membrane. Exp. Neurol..

[107] Weisova P., Concannon C.G., Devocelle M., Prehn J.H., Ward M.W. (2009). Regulation of glucose transporter 3 surface expression by the AMP-activated protein kinase mediates tolerance to glutamate excitation in neurons. J. Neurosci..

[108] Lin C.L., Cheng Y.S., Li H.H., Chiu P.Y., Chang Y.T., Ho Y.J., Lai T.J. (2016). Amyloid-beta suppresses AMP-activated protein kinase (AMPK) signaling and contributes to alpha-synuclein-induced cytotoxicity. Exp. Neurol..

[109] Chen M., Huang N., Liu J., Huang J., Shi J., Jin F. (2021). AMPK: A bridge between diabetes mellitus and Alzheimer’s disease. Behav. Brain Res..

[110] Winkler E.A., Nishida Y., Sagare A.P., Rege S.V., Bell R.D., Perlmutter D., Sengillo J.D., Hillman S., Kong P., Nelson A.R. (2015). GLUT1 reductions exacerbate Alzheimer’s disease vasculo-neuronal dysfunction and degeneration. Nat. Neurosci..

[111] Matioli M., Nitrini R. (2015). Mechanisms linking brain insulin resistance to Alzheimer’s disease. Dement. Neuropsychol..

[112] McNay E.C., Pearson-Leary J. (2020). GluT4: A central player in hippocampal memory and brain insulin resistance. Exp. Neurol..

[113] Liu W., Zhuo P., Li L., Jin H., Lin B., Zhang Y., Liang S., Wu J., Huang J., Wang Z. (2017). Activation of brain glucose metabolism ameliorating cognitive impairment in APP/PS1 transgenic mice by electroacupuncture. Free Radic. Biol. Med..

[114] Lee S., Tong M., Hang S., Deochand C., de la Monte S. (2013). CSF and Brain Indices of Insulin Resistance, Oxidative Stress and Neuro-Inflammation in Early versus Late Alzheimer’s Disease. J. Alzheimers Dis. Parkinsonism.

[115] Stanley M., Macauley S.L., Holtzman D.M. (2016). Changes in insulin and insulin signaling in Alzheimer’s disease: Cause or consequence?. J. Exp. Med..

[116] Wei Z., Koya J., Reznik S.E. (2021). Insulin Resistance Exacerbates Alzheimer Disease via Multiple Mechanisms. Front. Neurosci..

[117] Hoyer S., Nitsch R. (1989). Cerebral excess release of neurotransmitter amino acids subsequent to reduced cerebral glucose metabolism in early-onset dementia of Alzheimer type. J. Neural Transm..

[118] Hoyer S., Nitsch R., Oesterreich K. (1991). Predominant abnormality in cerebral glucose utilization in late-onset dementia of the Alzheimer type: A cross-sectional comparison against advanced late-onset and incipient early-onset cases. J. Neural Transm. Parkinson Dis. Dement. Sect..

[119] Rivera E.J., Goldin A., Fulmer N., Tavares R., Wands J.R., de la Monte S.M. (2005). Insulin and insulin-like growth factor expression and function deteriorate with progression of Alzheimer’s disease: Link to brain reductions in acetylcholine. J. Alzheimers Dis..

[120] Mosconi L., Pupi A., De Leon M.J. (2008). Brain glucose hypometabolism and oxidative stress in preclinical Alzheimer’s disease. Ann. N. Y. Acad. Sci..

[121] Langbaum J.B., Chen K., Caselli R.J., Lee W., Reschke C., Bandy D., Alexander G.E., Burns C.M., Kaszniak A.W., Reeder S.A. (2010). Hypometabolism in Alzheimer-affected brain regions in cognitively healthy Latino individuals carrying the apolipoprotein E ε4 allele. Arch. Neurol..

[122] Talbot K., Wang H.Y., Kazi H., Han L.Y., Bakshi K.P., Stucky A., Fuino R.L., Kawaguchi K.R., Samoyedny A.J., Wilson R.S. (2012). Demonstrated brain insulin resistance in Alzheimer’s disease patients is associated with IGF-1 resistance, IRS-1 dysregulation, and cognitive decline. J. Clin. Investig..

[123] Biessels G.J., Reagan L.P. (2015). Hippocampal insulin resistance and cognitive dysfunction. Nat. Rev. Neurosci..

[124] Cunnane S.C., Courchesne-Loyer A., St-Pierre V., Vandenberghe C., Pierotti T., Fortier M., Croteau E., Castellano C.A. (2016). Can ketones compensate for deteriorating brain glucose uptake during aging? Implications for the risk and treatment of Alzheimer’s disease. Ann. N. Y. Acad. Sci..

[125] Leissring M.A. (2021). Insulin-Degrading Enzyme: Paradoxes and Possibilities. Cells.

[126] Kurochkin I.V., Guarnera E., Berezovsky I.N. (2018). Insulin-Degrading Enzyme in the Fight against Alzheimer’s Disease. Trends Pharmacol. Sci..

[127] De la Monte S.M., Wands J.R. (2008). Alzheimer’s disease is type 3 diabetes-evidence reviewed. J. Diabetes Sci. Technol..

[128] Lewitt M.S., Boyd G.W. (2019). The Role of Insulin-Like Growth Factors and Insulin-Like Growth Factor-Binding Proteins in the Nervous System. Biochem. Insights.

[129] Kim B., Elzinga S.E., Henn R.E., McGinley L.M., Feldman E.L. (2019). The effects of insulin and insulin-like growth factor I on amyloid precursor protein phosphorylation in in vitro and in vivo models of Alzheimer’s disease. Neurobiol. Dis..

[130] Logan S., Pharaoh G.A., Marlin M.C., Masser D.R., Matsuzaki S., Wronowski B., Yeganeh A., Parks E.E., Premkumar P., Farley J.A. (2018). Insulin-like growth factor receptor signaling regulates working memory, mitochondrial metabolism, and amyloid-beta uptake in astrocytes. Mol. Metab..

[131] De la Monte S.M. (2012). Brain insulin resistance and deficiency as therapeutic targets in Alzheimer’s disease. Curr. Alzheimer Res..

[132] De la Monte S.M. (2012). Contributions of brain insulin resistance and deficiency in amyloid-related neurodegeneration in Alzheimer’s disease. Drugs.

[133] De la Monte S.M. (2017). Insulin Resistance and Neurodegeneration: Progress Towards the Development of New Therapeutics for Alzheimer’s Disease. Drugs.

[134] Hoyer S. (1996). Oxidative metabolism deficiencies in brains of patients with Alzheimer’s disease. Acta Neurol. Scand..

[135] Smith M.A., Perry G., Richey P.L., Sayre L.M., Anderson V.E., Beal M.F., Kowall N. (1996). Oxidative damage in Alzheimer’s. Nature.

[136] Rinaldi P., Polidori M.C., Metastasio A., Mariani E., Mattioli P., Cherubini A., Catani M., Cecchetti R., Senin U., Mecocci P. (2003). Plasma antioxidants are similarly depleted in mild cognitive impairment and in Alzheimer’s disease. Neurobiol. Aging..

[137] Butterfield D.A., Reed T., Newman S.F., Sultana R. (2007). Roles of amyloid beta-peptide-associated oxidative stress and brain protein modifications in the pathogenesis of Alzheimer’s disease and mild cognitive impairment. Free Radic. Biol. Med..

[138] Sultana R., Butterfield D.A. (2009). Oxidatively modified, mitochondria-relevant brain proteins in subjects with Alzheimer disease and mild cognitive impairment. J. Bioenerg. Biomembr..

[139] Butterfield D.A., Halliwell B. (2019). Oxidative stress, dysfunctional glucose metabolism and Alzheimer disease. Nat. Rev. Neurosci..

[140] Cenini G., Lloret A., Cascella R. (2019). Oxidative Stress in Neurodegenerative Diseases: From a Mitochondrial Point of View. Oxid. Med. Cell. Longev..

[141] Sharma C., Kim S.R. (2021). Linking Oxidative Stress and Proteinopathy in Alzheimer’s Disease. Antioxidants.

[142] Moreira P.I., Duarte A.I., Santos M.S., Rego A.C., Oliveira C.R. (2009). An integrative view of the role of oxidative stress, mitochondria and insulin in Alzheimer’s disease. J. Alzheimers Dis..

[143] Abeti R., Abramov A.Y., Duchen M.R. (2011). Beta-amyloid activates PARP causing astrocytic metabolic failure and neuronal death. Brain.

[144] Abeti R., Duchen M.R. (2012). Activation of PARP by oxidative stress induced by beta-amyloid: Implications for Alzheimer’s disease. Neurochem. Res..

[145] Cahill-Smith S., Li J.M. (2014). Oxidative stress, redox signalling and endothelial dysfunction in ageing-related neurodegenerative diseases: A role of NADPH oxidase 2. Br. J. Clin. Pharmacol..

[146] Pratico D., Uryu K., Leight S., Trojanoswki J.Q., Lee V.M. (2001). Increased lipid peroxidation precedes amyloid plaque formation in an animal model of Alzheimer amyloidosis. J. Neurosci..

[147] Petrovic S., Arsic A., Ristic-Medic D., Cvetkovic Z., Vucic V. (2020). Lipid Peroxidation and Antioxidant Supplementation in Neurodegenerative Diseases: A Review of Human Studies. Antioxidants.

[148] Angelova P.R., Esteras N., Abramov A.Y. (2021). Mitochondria and lipid peroxidation in the mechanism of neurodegeneration: Finding ways for prevention. Med. Res. Rev..

[149] Pena-Bautista C., Alvarez-Sanchez L., Ferrer I., Lopez-Nogueroles M., Canada-Martinez A.J., Oger C., Galano J.M., Durand T., Baquero M., Chafer-Pericas C. (2021). Lipid Peroxidation Assessment in Preclinical Alzheimer Disease Diagnosis. Antioxidants.

[150] Mark R.J., Pang Z., Geddes J.W., Uchida K., Mattson M.P. (1997). Amyloid beta-peptide impairs glucose transport in hippocampal and cortical neurons: Involvement of membrane lipid peroxidation. J. Neurosci..

[151] Gibson G.E., Starkov A., Blass J.P., Ratan R.R., Beal M.F. (2010). Cause and consequence: Mitochondrial dysfunction initiates and propagates neuronal dysfunction, neuronal death and behavioral abnormalities in age-associated neurodegenerative diseases. Biochim. Biophys. Acta.

[152] Friedland-Leuner K., Stockburger C., Denzer I., Eckert G.P., Muller W.E. (2014). Mitochondrial dysfunction: Cause and consequence of Alzheimer’s disease. Prog. Mol. Biol. Transl. Sci..

[153] Jove M., Mota-Martorell N., Torres P., Ayala V., Portero-Otin M., Ferrer I., Pamplona R. (2021). The Causal Role of Lipoxidative Damage in Mitochondrial Bioenergetic Dysfunction Linked to Alzheimer’s Disease Pathology. Life.

[154] Mancuso C., Bates T.E., Butterfield D.A., Calafato S., Cornelius C., De Lorenzo A., Dinkova Kostova A.T., Calabrese V. (2007). Natural antioxidants in Alzheimer’s disease. Expert Opin. Investig. Drugs.

[155] Wojsiat J., Zoltowska K.M., Laskowska-Kaszub K., Wojda U. (2018). Oxidant/Antioxidant Imbalance in Alzheimer’s Disease: Therapeutic and Diagnostic Prospects. Oxid. Med. Cell. Longev..

[156] Sinyor B., Mineo J., Ochner C. (2020). Alzheimer’s Disease, Inflammation, and the Role of Antioxidants. J. Alzheimers Dis. Rep..

[157] Fracassi A., Marcatti M., Zolochevska O., Tabor N., Woltjer R., Moreno S., Taglialatela G. (2021). Oxidative Damage and Antioxidant Response in Frontal Cortex of Demented and Nondemented Individuals with Alzheimer’s Neuropathology. J. Neurosci..

[158] Juszczyk G., Mikulska J., Kasperek K., Pietrzak D., Mrozek W., Herbet M. (2021). Chronic Stress and Oxidative Stress as Common Factors of the Pathogenesis of Depression and Alzheimer’s Disease: The Role of Antioxidants in Prevention and Treatment. Antioxidants.

[159] Sano M., Ernesto C., Thomas R.G., Klauber M.R., Schafer K., Grundman M., Woodbury P., Growdon J., Cotman C.W., Pfeiffer E. (1997). A controlled trial of selegiline, alpha-tocopherol, or both as treatment for Alzheimer’s disease. The Alzheimer’s Disease Cooperative Study. N. Engl. J. Med..

[160] Morris M.C., Beckett L.A., Scherr P.A., Hebert L.E., Bennett D.A., Field T.S., Evans D.A. (1998). Vitamin E and vitamin C supplement use and risk of incident Alzheimer disease. Alzheimer Dis Assoc. Disord..

[161] Morris M.C., Evans D.A., Bienias J.L., Tangney C.C., Bennett D.A., Aggarwal N., Wilson R.S., Scherr P.A. (2002). Dietary intake of antioxidant nutrients and the risk of incident Alzheimer disease in a biracial community study. JAMA.

[162] Luchsinger J.A., Tang M.X., Shea S., Mayeux R. (2003). Antioxidant vitamin intake and risk of Alzheimer disease. Arch. Neurol..

[163] Morris M.C., Evans D.A., Tangney C.C., Bienias J.L., Wilson R.S., Aggarwal N.T., Scherr P.A. (2005). Relation of the tocopherol forms to incident Alzheimer disease and to cognitive change. Am. J. Clin. Nutr..

[164] Petersen R.C., Thomas R.G., Grundman M., Bennett D., Doody R., Ferris S., Galasko D., Jin S., Kaye J., Levey A. (2005). Vitamin E and donepezil for the treatment of mild cognitive impairment. N. Engl. J. Med..

[165] Pham D.Q., Plakogiannis R. (2005). Vitamin E supplementation in Alzheimer’s disease, Parkinson’s disease, tardive dyskinesia, and cataract: Part 2. Ann. Pharmacother..

[166] DeKosky S.T., Williamson J.D., Fitzpatrick A.L., Kronmal R.A., Ives D.G., Saxton J.A., Lopez O.L., Burke G., Carlson M.C., Fried L.P. (2008). Ginkgo biloba for prevention of dementia: A randomized controlled trial. JAMA.

[167] Persson T., Popescu B.O., Cedazo-Minguez A. (2014). Oxidative stress in Alzheimer’s disease: Why did antioxidant therapy fail?. Oxid. Med. Cell. Longev..

[168] Halliwell B., Gutteridge J.M. (2007). Free Radicals in Biology and Medicine.

[169] Szeto H.H. (2006). Cell-permeable, mitochondrial-targeted, peptide antioxidants. AAPS J..

[170] Szeto H.H. (2006). Mitochondria-targeted peptide antioxidants: Novel neuroprotective agents. AAPS J..

[171] Sheu S.S., Nauduri D., Anders M.W. (2006). Targeting antioxidants to mitochondria: A new therapeutic direction. Biochim. Biophys. Acta.

[172] Reddy P.H. (2006). Mitochondrial oxidative damage in aging and Alzheimer’s disease: Implications for mitochondrially targeted antioxidant therapeutics. J. Biomed. Biotechnol..

[173] Murphy M.P., Smith R.A. (2007). Targeting antioxidants to mitochondria by conjugation to lipophilic cations. Annu. Rev. Pharmacol. Toxicol..

[174] Szeto H.H. (2008). Development of mitochondria-targeted aromatic-cationic peptides for neurodegenerative diseases. Ann. N. Y. Acad. Sci..

[175] Reddy P.H. (2008). Mitochondrial medicine for aging and neurodegenerative diseases. Neuromol. Med..

[176] Murphy M.P. (1997). Selective targeting of bioactive compounds to mitochondria. Trends Biotechnol..

[177] Kelso G.F., Porteous C.M., Coulter C.V., Hughes G., Porteous W.K., Ledgerwood E.C., Smith R.A., Murphy M.P. (2001). Selective targeting of a redox-active ubiquinone to mitochondria within cells: Antioxidant and antiapoptotic properties. J. Biol. Chem..

[178] James A.M., Sharpley M.S., Manas A.R., Frerman F.E., Hirst J., Smith R.A., Murphy M.P. (2007). Interaction of the mitochondria-targeted antioxidant MitoQ with phospholipid bilayers and ubiquinone oxidoreductases. J. Biol. Chem..

[179] McManus M.J., Murphy M.P., Franklin J.L. (2011). The mitochondria-targeted antioxidant MitoQ prevents loss of spatial memory retention and early neuropathology in a transgenic mouse model of Alzheimer’s disease. J. Neurosci..

[180] Young M.L., Franklin J.L. (2019). The mitochondria-targeted antioxidant MitoQ inhibits memory loss, neuropathology, and extends lifespan in aged 3xTg-AD mice. Mol. Cell Neurosci..

[181] Manczak M., Mao P., Calkins M.J., Cornea A., Reddy A.P., Murphy M.P., Szeto H.H., Park B., Reddy P.H. (2010). Mitochondria-targeted antioxidants protect against amyloid-beta toxicity in Alzheimer’s disease neurons. J. Alzheimers Dis..

[182] Ng L.F., Gruber J., Cheah I.K., Goo C.K., Cheong W.F., Shui G., Sit K.P., Wenk M.R., Halliwell B. (2014). The mitochondria-targeted antioxidant MitoQ extends lifespan and improves healthspan of a transgenic *Caenorhabditis elegans* model of Alzheimer disease. Free Radic. Biol. Med..

[183] Snow B.J., Rolfe F.L., Lockhart M.M., Frampton C.M., O’Sullivan J.D., Fung V., Smith R.A., Murphy M.P., Taylor K.M., Protect Study G. (2010). A double-blind, placebo-controlled study to assess the mitochondria-targeted antioxidant MitoQ as a disease-modifying therapy in Parkinson’s disease. Mov. Disord..

[184] Schiller P.W., Nguyen T.M., Berezowska I., Dupuis S., Weltrowska G., Chung N.N., Lemieux C. (2000). Synthesis and in vitro opioid activity profiles of DALDA analogues. Eur. J. Med. Chem..

[185] Zhao K., Zhao G.M., Wu D., Soong Y., Birk A.V., Schiller P.W., Szeto H.H. (2004). Cell-permeable peptide antioxidants targeted to inner mitochondrial membrane inhibit mitochondrial swelling, oxidative cell death, and reperfusion injury. J. Biol. Chem..

[186] Reddy P.H., Manczak M., Kandimalla R. (2017). Mitochondria-targeted small molecule SS31: A potential candidate for the treatment of Alzheimer’s disease. Hum. Mol. Genet..

[187] Ding X.W., Robinson M., Li R., Aldhowayan H., Geetha T., Babu J.R. (2021). Mitochondrial dysfunction and beneficial effects of mitochondria-targeted small peptide SS-31 in Diabetes Mellitus and Alzheimer’s disease. Pharmacol. Res..

[188] Zhao W., Xu Z., Cao J., Fu Q., Wu Y., Zhang X., Long Y., Zhang X., Yang Y., Li Y. (2019). Elamipretide (SS-31) improves mitochondrial dysfunction, synaptic and memory impairment induced by lipopolysaccharide in mice. J. Neuroinflamm..

[189] Liu Y., Fu H., Wu Y., Nie B., Liu F., Wang T., Xiao W., Yang S., Kan M., Fan L. (2021). Elamipretide (SS-31) Improves Functional Connectivity in Hippocampus and Other Related Regions Following Prolonged Neuroinflammation Induced by Lipopolysaccharide in Aged Rats. Front. Aging Neurosci..

[190] Skulachev M.V., Antonenko Y.N., Anisimov V.N., Chernyak B.V., Cherepanov D.A., Chistyakov V.A., Egorov M.V., Kolosova N.G., Korshunova G.A., Lyamzaev K.G. (2011). Mitochondrial-targeted plastoquinone derivatives. Effect on senescence and acute age-related pathologies. Curr. Drug Targets.

[191] Isaev N.K., Stelmashook E.V., Genrikhs E.E., Korshunova G.A., Sumbatyan N.V., Kapkaeva M.R., Skulachev V.P. (2016). Neuroprotective properties of mitochondria-targeted antioxidants of the SkQ-type. Rev. Neurosci..

[192] Shabalina I.G., Vyssokikh M.Y., Gibanova N., Csikasz R.I., Edgar D., Hallden-Waldemarson A., Rozhdestvenskaya Z., Bakeeva L.E., Vays V.B., Pustovidko A.V. (2017). Improved health-span and lifespan in mtDNA mutator mice treated with the mitochondrially targeted antioxidant SkQ1. Aging.

[193] Salganik R.I., Solovyova N.A., Dikalov S.I., Grishaeva O.N., Semenova L.A., Popovsky A.V. (1994). Inherited enhancement of hydroxyl radical generation and lipid peroxidation in the S strain rats results in DNA rearrangements, degenerative diseases, and premature aging. Biochem. Biophys. Res. Commun..

[194] Stelmashook E.V., Stavrovskaya A.V., Yamshchikova N.G., Ol’shanskii A.S., Kapay N.A., Popova O.V., Khaspekov L.G., Skrebitsky V.G., Isaev N.K. (2015). Mitochondria-Targeted Plastoquinone Antioxidant SkQR1 Has Positive Effect on Memory of Rats. Biochemistry.

[195] Loshchenova P.S., Sinitsyna O.I., Fedoseeva L.A., Stefanova N.A., Kolosova N.G. (2015). Influence of Antioxidant SkQ1 on Accumulation of Mitochondrial DNA Deletions in the Hippocampus of Senescence-Accelerated OXYS Rats. Biochemistry.

[196] Skulachev M.V., Skulachev V.P. (2017). Programmed Aging of Mammals: Proof of Concept and Prospects of Biochemical Approaches for Anti-aging Therapy. Biochemistry.

[197] Stefanova N.A., Muraleva N.A., Maksimova K.Y., Rudnitskaya E.A., Kiseleva E., Telegina D.V., Kolosova N.G. (2016). An antioxidant specifically targeting mitochondria delays progression of Alzheimer’s disease-like pathology. Aging.

[198] Stefanova N.A., Muraleva N.A., Skulachev V.P., Kolosova N.G. (2014). Alzheimer’s disease-like pathology in senescence-accelerated OXYS rats can be partially retarded with mitochondria-targeted antioxidant SkQ1. J. Alzheimers Dis..

[199] Kolosova N.G., Tyumentsev M.A., Muraleva N.A., Kiseleva E., Vitovtov A.O., Stefanova N.A. (2017). Antioxidant SkQ1 Alleviates Signs of Alzheimer’s Disease-like Pathology in Old OXYS Rats by Reversing Mitochondrial Deterioration. Curr. Alzheimer Res..

[200] Stefanova N.A., Ershov N.I., Kolosova N.G. (2019). Suppression of Alzheimer’s Disease-Like Pathology Progression by Mitochondria-Targeted Antioxidant SkQ1: A Transcriptome Profiling Study. Oxid. Med. Cell. Longev..

[201] Sukhorukov V.S., Mudzhiri N.M., Voronkova A.S., Baranich T.I., Glinkina V.V., Illarioshkin S.N. (2021). Mitochondrial Disorders in Alzheimer’s Disease. Biochemistry.

[202] Kapay N.A., Isaev N.K., Stelmashook E.V., Popova O.V., Zorov D.B., Skrebitsky V.G., Skulachev V.P. (2011). In vivo injected mitochondria-targeted plastoquinone antioxidant SkQR1 prevents beta-amyloid-induced decay of long-term potentiation in rat hippocampal slices. Biochemistry.

[203] Genrikhs E.E., Stelmashook E.V., Popova O.V., Kapay N.A., Korshunova G.A., Sumbatyan N.V., Skrebitsky V.G., Skulachev V.P., Isaev N.K. (2015). Mitochondria-targeted antioxidant SkQT1 decreases trauma-induced neurological deficit in rat and prevents amyloid-beta-induced impairment of long-term potentiation in rat hippocampal slices. J. Drug Target..

[204] Smith R.A., Porteous C.M., Coulter C.V., Murphy M.P. (1999). Selective targeting of an antioxidant to mitochondria. Eur. J. Biochem..

[205] Smith R.A., Porteous C.M., Gane A.M., Murphy M.P. (2003). Delivery of bioactive molecules to mitochondria in vivo. Proc. Natl. Acad. Sci. USA.

[206] Zang Q.S., Sadek H., Maass D.L., Martinez B., Ma L., Kilgore J.A., Williams N.S., Frantz D.E., Wigginton J.G., Nwariaku F.E. (2012). Specific inhibition of mitochondrial oxidative stress suppresses inflammation and improves cardiac function in a rat pneumonia-related sepsis model. Am. J. Physiol. Heart Circ. Physiol..

[207] Lowes D.A., Webster N.R., Murphy M.P., Galley H.F. (2013). Antioxidants that protect mitochondria reduce interleukin-6 and oxidative stress, improve mitochondrial function, and reduce biochemical markers of organ dysfunction in a rat model of acute sepsis. Br. J. Anaesth..

[208] McCormick B., Lowes D.A., Colvin L., Torsney C., Galley H.F. (2016). MitoVitE, a mitochondria-targeted antioxidant, limits paclitaxel-induced oxidative stress and mitochondrial damage in vitro, and paclitaxel-induced mechanical hypersensitivity in a rat pain model. Br. J. Anaesth..

[209] Minter B.E., Lowes D.A., Webster N.R., Galley H.F. (2020). Differential Effects of MitoVitE, alpha-Tocopherol and Trolox on Oxidative Stress, Mitochondrial Function and Inflammatory Signalling Pathways in Endothelial Cells Cultured under Conditions Mimicking Sepsis. Antioxidants.

[210] Macia E., Ehrlich M., Massol R., Boucrot E., Brunner C., Kirchhausen T. (2006). Dynasore, a cell-permeable inhibitor of dynamin. Dev. Cell..

[211] Misrani A., Tabassum S., Yang L. (2021). Mitochondrial Dysfunction and Oxidative Stress in Alzheimer’s Disease. Front. Aging Neurosci..

[212] Trnka J., Blaikie F.H., Smith R.A., Murphy M.P. (2008). A mitochondria-targeted nitroxide is reduced to its hydroxylamine by ubiquinol in mitochondria. Free Radic. Biol. Med..

[213] Hu H., Li M. (2016). Mitochondria-targeted antioxidant mitotempo protects mitochondrial function against amyloid beta toxicity in primary cultured mouse neurons. Biochem. Biophys. Res. Commun..

[214] Zhan L., Li R., Sun Y., Dou M., Yang W., He S., Zhang Y. (2018). Effect of mito-TEMPO, a mitochondria-targeted antioxidant, in rats with neuropathic pain. NeuroReport.

[215] Ni R., Cao T., Xiong S., Ma J., Fan G.C., Lacefield J.C., Lu Y., Le Tissier S., Peng T. (2016). Therapeutic inhibition of mitochondrial reactive oxygen species with mito-TEMPO reduces diabetic cardiomyopathy. Free Radic. Biol. Med..

[216] Zhang J., Wang Q., Xu C., Lu Y., Hu H., Qin B., Wang Y., He D., Li C., Yu X. (2017). MitoTEMPO Prevents Oxalate Induced Injury in NRK-52E Cells via Inhibiting Mitochondrial Dysfunction and Modulating Oxidative Stress. Oxid. Med. Cell. Longev..

[217] Arancio O., Zhang H.P., Chen X., Lin C., Trinchese F., Puzzo D., Liu S., Hegde A., Yan S.F., Stern A. (2004). RAGE potentiates Aβ-induced perturbation of neuronal function in transgenic mice. EMBO J..

[218] Guo L., Du H., Yan S., Wu X., McKhann G.M., Chen J.X., Yan S.S. (2013). Cyclophilin D deficiency rescues axonal mitochondrial transport in Alzheimer’s neurons. PLoS ONE.

[219] Gan X., Huang S., Wu L., Wang Y., Hu G., Li G., Zhang H., Yu H., Swerdlow R.H., Chen J.X. (2014). Inhibition of ERK-DLP1 signaling and mitochondrial division alleviates mitochondrial dysfunction in Alzheimer’s disease cybrid cell. Biochim. Biophys. Acta.

[220] Falcicchia C., Tozzi F., Arancio O., Watterson D.M., Origlia N. (2020). Involvement of p38 MAPK in Synaptic Function and Dysfunction. Int. J. Mol. Sci..

[221] Yu Q., Wang Y., Du F., Yan S., Hu G., Origlia N., Rutigliano G., Sun Q., Yu H., Ainge J. (2018). Overexpression of endophilin A1 exacerbates synaptic alterations in a mouse model of Alzheimer’s disease. Nat. Commun..

[222] Mukem S., Thongbuakaew T., Khornchatri K. (2021). Mito-Tempo suppresses autophagic flux via the PI3K/Akt/mTOR signaling pathway in neuroblastoma SH-SY5Y cells. Heliyon.

[223] Langley M., Ghosh A., Charli A., Sarkar S., Ay M., Luo J., Zielonka J., Brenza T., Bennett B., Jin H. (2017). Mito-Apocynin Prevents Mitochondrial Dysfunction, Microglial Activation, Oxidative Damage, and Progressive Neurodegeneration in MitoPark Transgenic Mice. Antioxid. Redox Signal..

[224] Dranka B.P., Gifford A., McAllister D., Zielonka J., Joseph J., O’Hara C.L., Stucky C.L., Kanthasamy A.G., Kalyanaraman B. (2014). A novel mitochondrially-targeted apocynin derivative prevents hyposmia and loss of motor function in the leucine-rich repeat kinase 2 (LRRK2(R1441G)) transgenic mouse model of Parkinson’s disease. Neurosci. Lett..

[225] Ghosh A., Langley M.R., Harischandra D.S., Neal M.L., Jin H., Anantharam V., Joseph J., Brenza T., Narasimhan B., Kanthasamy A. (2016). Mitoapocynin Treatment Protects Against Neuroinflammation and Dopaminergic Neurodegeneration in a Preclinical Animal Model of Parkinson’s Disease. J. Neuroimmune Pharmacol..

[226] Liu N., Lin M.M., Huang S.S., Liu Z.Q., Wu J.C., Liang Z.Q., Qin Z.H., Wang Y. (2021). NADPH and Mito-Apocynin Treatment Protects Against KA-Induced Excitotoxic Injury Through Autophagy Pathway. Front. Cell Dev. Biol..

[227] Ross K.A., Brenza T.M., Binnebose A.M., Phanse Y., Kanthasamy A.G., Gendelman H.E., Salem A.K., Bartholomay L.C., Bellaire B.H., Narasimhan B. (2015). Nano-enabled delivery of diverse payloads across complex biological barriers. J. Control. Release.

[228] Mallapragada S.K., Brenza T.M., McMillan J.M., Narasimhan B., Sakaguchi D.S., Sharma A.D., Zbarska S., Gendelman H.E. (2015). Enabling nanomaterial, nanofabrication and cellular technologies for nanoneuromedicines. Nanomedicine.

[229] Binda A., Murano C., Rivolta I. (2020). Innovative Therapies and Nanomedicine Applications for the Treatment of Alzheimer’s Disease: A State-of-the-Art (2017–2020). Int. J. Nanomed..

[230] Brenza T.M., Ghaisas S., Ramirez J.E.V., Harischandra D., Anantharam V., Kalyanaraman B., Kanthasamy A.G., Narasimhan B. (2017). Neuronal protection against oxidative insult by polyanhydride nanoparticle-based mitochondria-targeted antioxidant therapy. Nanomedicine.

[231] Mahmood A., Bisoyi P., Banerjee R., Yousuf M., Goswami S.K. (2021). Mitoapocynin, a mitochondria targeted derivative of apocynin induces mitochondrial ROS generation and apoptosis in multiple cell types including cardiac myoblasts: A potential constraint to its therapeutic use. Mol. Cell Biochem..

[232] Reddy P.H. (2009). Amyloid beta, mitochondrial structural and functional dynamics in Alzheimer’s disease. Exp. Neurol..

[233] Manczak M., Calkins M.J., Reddy P.H. (2011). Impaired mitochondrial dynamics and abnormal interaction of amyloid beta with mitochondrial protein Drp1 in neurons from patients with Alzheimer’s disease: Implications for neuronal damage. Hum. Mol. Genet..

[234] Manczak M., Reddy P.H. (2012). Abnormal interaction between the mitochondrial fission protein Drp1 and hyperphosphorylated tau in Alzheimer’s disease neurons: Implications for mitochondrial dysfunction and neuronal damage. Hum. Mol. Genet..

[235] Manczak M., Kandimalla R., Yin X., Reddy P.H. (2018). Hippocampal mutant APP and amyloid beta-induced cognitive decline, dendritic spine loss, defective autophagy, mitophagy and mitochondrial abnormalities in a mouse model of Alzheimer’s disease. Hum. Mol. Genet..

[236] Cassidy-Stone A., Chipuk J.E., Ingerman E., Song C., Yoo C., Kuwana T., Kurth M.J., Shaw J.T., Hinshaw J.E., Green D. (2008). Ret al. Chemical inhibition of the mitochondrial division dynamin reveals its role in Bax/Bak-dependent mitochondrial outer membrane permeabilization. Dev. Cell.

[237] Manczak M., Kandimalla R., Yin X., Reddy P.H. (2019). Mitochondrial division inhibitor 1 reduces dynamin-related protein 1 and mitochondrial fission activity. Hum. Mol. Genet..

[238] Baek S.H., Park S.J., Jeong J.I., Kim S.H., Han J., Kyung J.W., Baik S.H., Choi Y., Choi B.Y., Park J.S. (2017). Inhibition of Drp1 Ameliorates Synaptic Depression, Abeta Deposition, and Cognitive Impairment in an Alzheimer’s Disease Model. J. Neurosci..

[239] Wang W., Yin J., Ma X., Zhao F., Siedlak S.L., Wang Z., Torres S., Fujioka H., Xu Y., Perry G. (2017). Inhibition of mitochondrial fragmentation protects against Alzheimer’s disease in rodent model. Hum. Mol. Genet..

[240] Reddy P.H., Manczak M., Yin X. (2017). Mitochondria-Division Inhibitor 1 Protects Against Amyloid-beta induced Mitochondrial Fragmentation and Synaptic Damage in Alzheimer’s Disease. J. Alzheimers Dis..

[241] Reddy P.H., Manczak M., Yin X., Reddy A.P. (2018). Synergistic Protective Effects of Mitochondrial Division Inhibitor 1 and Mitochondria-Targeted Small Peptide SS31 in Alzheimer’s Disease. J. Alzheimers Dis..

[242] Bido S., Soria F.N., Fan R.Z., Bezard E., Tieu K. (2017). Mitochondrial division inhibitor-1 is neuroprotective in the A53T-alpha-synuclein rat model of Parkinson’s disease. Sci. Rep..

[243] Bordt E.A., Clerc P., Roelofs B.A., Saladino A.J., Tretter L., Adam-Vizi V., Cherok E., Khalil A., Yadava N., Ge S.X. (2017). The Putative Drp1 Inhibitor mdivi-1 Is a Reversible Mitochondrial Complex I Inhibitor that Modulates Reactive Oxygen Species. Dev. Cell..

[244] Satoi H., Tomimoto H., Ohtani R., Kitano T., Kondo T., Watanabe M., Oka N., Akiguchi I., Furuya S., Hirabayashi Y. (2005). Astroglial expression of ceramide in Alzheimer’s disease brains: A role during neuronal apoptosis. Neuroscience.

[245] McGrath E.R., Himali J.J., Xanthakis V., Duncan M.S., Schaffer J.E., Ory D.S., Peterson L.R., DeCarli C., Pase M.P., Satizabal C.L. (2020). Circulating ceramide ratios and risk of vascular brain aging and dementia. Ann. Clin. Transl. Neurol..

[246] Han X., Holtzman D.M., McKeel D.W., Kelley J., Morris J.C. (2002). Substantial sulfatide deficiency and ceramide elevation in very early Alzheimer’s disease: Potential role in disease pathogenesis. J. Neurochem..

[247] Filippov V., Song M.A., Zhang K., Vinters H.V., Tung S., Kirsch W.M., Yang J., Duerksen-Hughes P.J. (2012). Increased ceramide in brains with Alzheimer’s and other neurodegenerative diseases. J. Alzheimers Dis..

[248] Kim M., Nevado-Holgado A., Whiley L., Snowden S.G., Soininen H., Kloszewska I., Mecocci P., Tsolaki M., Vellas B., Thambisetty M. (2017). Association between Plasma Ceramides and Phosphatidylcholines and Hippocampal Brain Volume in Late Onset Alzheimer’s Disease. J. Alzheimers Dis..

[249] Byeon S.K., Madugundu A.K., Jain A.P., Bhat F.A., Jung J.H., Renuse S., Darrow J., Bakker A., Albert M., Moghekar A. (2021). Cerebrospinal fluid lipidomics for biomarkers of Alzheimer’s disease. Mol. Omics.

[250] Puglielli L., Ellis B.C., Saunders A.J., Kovacs D.M. (2003). Ceramide stabilizes beta-site amyloid precursor protein-cleaving enzyme 1 and promotes amyloid beta-peptide biogenesis. J. Biol. Chem..

[251] Crivelli S.M., Luo Q., Stevens J.A.A., Giovagnoni C., van Kruining D., Bode G., den Hoedt S., Hobo B., Scheithauer A.L., Walter J. (2021). CERTL reduces C16 ceramide, amyloid-beta levels, and inflammation in a model of Alzheimer’s disease. Alzheimers Res. Ther..

[252] Elsherbini A., Kirov A.S., Dinkins M.B., Wang G., Qin H., Zhu Z., Tripathi P., Crivelli S.M., Bieberich E. (2020). Association of Abeta with ceramide-enriched astrosomes mediates Abeta neurotoxicity. Acta Neuropathol. Commun..

[253] Jayashankar V., Selwan E., Hancock S.E., Verlande A., Goodson M.O., Eckenstein K.H., Milinkeviciute G., Hoover B.M., Chen B., Fleischman A.G. (2021). Drug-like sphingolipid SH-BC-893 opposes ceramide-induced mitochondrial fission and corrects diet-induced obesity. EMBO Mol. Med..

[254] Muley C., Bartelt A. (2021). Fuse your mitochondria, lose appetite: An anorexic, anti-obesity sphingolipid. EMBO Mol. Med..

[255] Guo X., Disatnik M.H., Monbureau M., Shamloo M., Mochly-Rosen D., Qi X. (2013). Inhibition of mitochondrial fragmentation diminishes Huntington’s disease-associated neurodegeneration. J. Clin. Investig..

[256] Qi X., Qvit N., Su Y.C., Mochly-Rosen D. (2013). A novel Drp1 inhibitor diminishes aberrant mitochondrial fission and neurotoxicity. J. Cell Sci..

[257] Numadate A., Mita Y., Matsumoto Y., Fujii S., Hashimoto Y. (2014). Development of 2-thioxoquinazoline-4-one derivatives as dual and selective inhibitors of dynamin-related protein 1 (Drp1) and puromycin-sensitive aminopeptidase (PSA). Chem. Pharm. Bull..

[258] Mallat A., Uchiyama L.F., Lewis S.C., Fredenburg R.A., Terada Y., Ji N., Nunnari J., Tseng C.C. (2018). Discovery and characterization of selective small molecule inhibitors of the mammalian mitochondrial division dynamin, DRP1. Biochem. Biophys. Res. Commun..

[259] Kuruva C.S., Manczak M., Yin X., Ogunmokun G., Reddy A.P., Reddy P.H. (2017). Aqua-soluble DDQ reduces the levels of Drp1 and Aβ and inhibits abnormal interactions between Aβ and Drp1 and protects Alzheimer’s disease neurons from Abeta- and Drp1-induced mitochondrial and synaptic toxicities. Hum. Mol. Genet..

[260] Van Praag H., Christie B.R., Sejnowski T.J., Gage F.H. (1999). Running enhances neurogenesis, learning, and long-term potentiation in mice. Proc. Natl. Acad. Sci. USA.

[261] Lee J., Duan W., Mattson M.P. (2002). Evidence that brain-derived neurotrophic factor is required for basal neurogenesis and mediates, in part, the enhancement of neurogenesis by dietary restriction in the hippocampus of adult mice. J. Neurochem..

[262] Navarro A., Gomez C., Lopez-Cepero J.M., Boveris A. (2004). Beneficial effects of moderate exercise on mice aging: Survival, behavior, oxidative stress, and mitochondrial electron transfer. Am. J. Physiol. Regul. Integr. Comp. Physiol..

[263] Stranahan A.M., Lee K., Martin B., Maudsley S., Golden E., Cutler R.G., Mattson M.P. (2009). Voluntary exercise and caloric restriction enhance hippocampal dendritic spine density and BDNF levels in diabetic mice. Hippocampus.

[264] Liu J., Yeo H.C., Overvik-Douki E., Hagen T., Doniger S.J., Chyu D.W., Brooks G.A., Ames B.N. (2000). Chronically and acutely exercised rats: Biomarkers of oxidative stress and endogenous antioxidants. J. Appl. Physiol..

[265] Quan H., Koltai E., Suzuki K., Aguiar A.S., Pinho R., Boldogh I., Berkes I., Radak Z. (2020). Exercise, redox system and neurodegenerative diseases. Biochim. Biophys. Acta Mol. Basis Dis..

[266] Liu H.L., Zhao G., Cai K., Zhao H.H., Shi L.D. (2011). Treadmill exercise prevents decline in spatial learning and memory in APP/PS1 transgenic mice through improvement of hippocampal long-term potentiation. Behav. Brain Res..

[267] Liu H.L., Zhao G., Zhang H., Shi L.D. (2013). Long-term treadmill exercise inhibits the progression of Alzheimer’s disease-like neuropathology in the hippocampus of APP/PS1 transgenic mice. Behav. Brain Res..

[268] Zhao G., Liu H.L., Zhang H., Tong X.J. (2015). Treadmill exercise enhances synaptic plasticity, but does not alter beta-amyloid deposition in hippocampi of aged APP/PS1 transgenic mice. Neuroscience.

[269] Colcombe S., Kramer A.F. (2003). Fitness effects on the cognitive function of older adults: A meta-analytic study. Psychol. Sci..

[270] Colcombe S.J., Erickson K.I., Scalf P.E., Kim J.S., Prakash R., McAuley E., Elavsky S., Marquez D.X., Hu L., Kramer A.F. (2006). Aerobic exercise training increases brain volume in aging humans. J. Gerontol. A Biol. Sci. Med. Sci..

[271] Smith P.J., Blumenthal J.A., Hoffman B.M., Cooper H., Strauman T.A., Welsh-Bohmer K., Browndyke J.N., Sherwood A. (2010). Aerobic exercise and neurocognitive performance: A meta-analytic review of randomized controlled trials. Psychosom. Med..

[272] Mattson M.P. (2012). Energy intake and exercise as determinants of brain health and vulnerability to injury and disease. Cell Metab..

[273] Curlik D.M., Shors T.J. (2013). Training your brain: Do mental and physical (MAP) training enhance cognition through the process of neurogenesis in the hippocampus?. Neuropharmacology.

[274] Kramer A.F., Colcombe S. (2018). Fitness Effects on the Cognitive Function of Older Adults: A Meta-Analytic Study-Revisited. Perspect. Psychol. Sci..

[275] Stern Y., MacKay-Brandt A., Lee S., McKinley P., McIntyre K., Razlighi Q., Agarunov E., Bartels M., Sloan R.P. (2019). Effect of aerobic exercise on cognition in younger adults: A randomized clinical trial. Neurology.

[276] Castells-Sanchez A., Roig-Coll F., Dacosta-Aguayo R., Lamonja-Vicente N., Sawicka A.K., Toran-Monserrat P., Pera G., Montero-Alia P., Heras-Tebar A., Domenech S. (2021). Exercise and Fitness Neuroprotective Effects: Molecular, Brain Volume and Psychological Correlates and Their Mediating Role in Healthy Late-Middle-Aged Women and Men. Front. Aging Neurosci..

[277] Erickson K.I., Prakash R.S., Voss M.W., Chaddock L., Hu L., Morris K.S., White S.M., Wojcicki T.R., McAuley E., Kramer A.F. (2009). Aerobic fitness is associated with hippocampal volume in elderly humans. Hippocampus.

[278] Erickson K.I., Voss M.W., Prakash R.S., Basak C., Szabo A., Chaddock L., Kim J.S., Heo S., Alves H., White S.M. (2011). Exercise training increases size of hippocampus and improves memory. Proc. Natl. Acad. Sci. USA.

[279] Berchicci M., Lucci G., Di Russo F. (2013). Benefits of physical exercise on the aging brain: The role of the prefrontal cortex. J. Gerontol. A Biol. Sci. Med. Sci..

[280] Erickson K.I., Leckie R.L., Weinstein A.M. (2014). Physical activity, fitness, and gray matter volume. Neurobiol. Aging.

[281] Valenzuela P.L., Castillo-Garcia A., Morales J.S., de la Villa P., Hampel H., Emanuele E., Lista S., Lucia A. (2020). Exercise benefits on Alzheimer’s disease: State-of-the-science. Ageing Res. Rev..

[282] Marques-Aleixo I., Beleza J., Sampaio A., Stevanovic J., Coxito P., Goncalves I., Ascensao A., Magalhaes J. (2021). Preventive and Therapeutic Potential of Physical Exercise in Neurodegenerative Diseases. Antioxid. Redox Signal..

[283] Puente-Gonzalez A.S., Sanchez-Sanchez M.C., Fernandez-Rodriguez E.J., Hernandez-Xumet J.E., Barbero-Iglesias F.J., Mendez-Sanchez R. (2021). Effects of 6-Month Multimodal Physical Exercise Program on Bone Mineral Density, Fall Risk, Balance, and Gait in Patients with Alzheimer’s Disease: A Controlled Clinical Trial. Brain Sci..

[284] Lopez-Ortiz S., Pinto-Fraga J., Valenzuela P.L., Martin-Hernandez J., Seisdedos M.M., Garcia-Lopez O., Toschi N., Di Giuliano F., Garaci F., Mercuri N.B. (2021). Physical Exercise and Alzheimer’s Disease: Effects on Pathophysiological Molecular Pathways of the Disease. Int. J. Mol. Sci..

[285] Tan Z.X., Dong F., Wu L.Y., Feng Y.S., Zhang F. (2021). The Beneficial Role of Exercise on Treating Alzheimer’s Disease by Inhibiting β-Amyloid Peptide. Mol. Neurobiol..

[286] Memme J.M., Erlich A.T., Phukan G., Hood D.A. (2021). Exercise and mitochondrial health. J. Physiol..

[287] Oliveira A.N., Richards B.J., Slavin M., Hood D.A. (2021). Exercise Is Muscle Mitochondrial Medicine. Exerc. Sport Sci. Rev..

[288] Broskey N.T., Greggio C., Boss A., Boutant M., Dwyer A., Schlueter L., Hans D., Gremion G., Kreis R., Boesch C. (2014). Skeletal muscle mitochondria in the elderly: Effects of physical fitness and exercise training. J. Clin. Endocrinol. Metab..

[289] Pedersen B.K. (2019). Physical activity and muscle-brain crosstalk. Nat. Rev. Endocrinol..

[290] Burtscher J., Burtscher M. (2020). Run for your life: Tweaking the weekly physical activity volume for longevity. Br. J. Sports Med..

[291] Burtscher J., Millet G.P., Place N., Kayser B., Zanou N. (2021). The Muscle-Brain Axis and Neurodegenerative Diseases: The Key Role of Mitochondria in Exercise-Induced Neuroprotection. Int. J. Mol. Sci..

[292] Hoffmann K., Sobol N.A., Frederiksen K.S., Beyer N., Vogel A., Vestergaard K., Braendgaard H., Gottrup H., Lolk A., Wermuth L. (2016). Moderate-to-High Intensity Physical Exercise in Patients with Alzheimer’s Disease: A Randomized Controlled Trial. J. Alzheimers Dis..

[293] Sobol N.A., Hoffmann K., Frederiksen K.S., Vogel A., Vestergaard K., Braendgaard H., Gottrup H., Lolk A., Wermuth L., Jakobsen S. (2016). Effect of aerobic exercise on physical performance in patients with Alzheimer’s disease. Alzheimers Dement..

[294] Sobol N.A., Dall C.H., Hogh P., Hoffmann K., Frederiksen K.S., Vogel A., Siersma V., Waldemar G., Hasselbalch S.G., Beyer N. (2018). Change in Fitness and the Relation to Change in Cognition and Neuropsychiatric Symptoms After Aerobic Exercise in Patients with Mild Alzheimer’s Disease. J. Alzheimers Dis..

[295] Morris J.K., Vidoni E.D., Johnson D.K., Van Sciver A., Mahnken J.D., Honea R.A., Wilkins H.M., Brooks W.M., Billinger S.A., Swerdlow R.H. (2017). Aerobic exercise for Alzheimer’s disease: A randomized controlled pilot trial. PLoS ONE.

[296] Yu F., Vock D.M., Zhang L., Salisbury D., Nelson N.W., Chow L.S., Smith G., Barclay T.R., Dysken M., Wyman J.F. (2021). Cognitive Effects of Aerobic Exercise in Alzheimer’s Disease: A Pilot Randomized Controlled Trial. J. Alzheimers Dis..

[297] Jia R.X., Liang J.H., Xu Y., Wang Y.Q. (2019). Effects of physical activity and exercise on the cognitive function of patients with Alzheimer disease: A meta-analysis. BMC Geriatr..

[298] De Farias J.M., Dos Santos Tramontin N., Pereira E.V., de Moraes G.L., Furtado B.G., Tietbohl L.T.W., Da Costa Pereira B., Simon K.U., Muller A.P. (2021). Physical Exercise Training Improves Judgment and Problem-Solving and Modulates Serum Biomarkers in Patients with Alzheimer’s Disease. Mol. Neurobiol..

[299] Zheng G., Xia R., Zhou W., Tao J., Chen L. (2016). Aerobic exercise ameliorates cognitive function in older adults with mild cognitive impairment: A systematic review and meta-analysis of randomised controlled trials. Br. J. Sports Med..

[300] Cammisuli D.M., Innocenti A., Fusi J., Franzoni F., Pruneti C. (2018). Aerobic exercise effects upon cognition in Alzheimer’s Disease: A systematic review of randomized controlled trials. Arch. Ital. Biol..

[301] Zhu Y., Zhong Q., Ji J., Ma J., Wu H., Gao Y., Ali N., Wang T. (2020). Effects of Aerobic Dance on Cognition in Older Adults with Mild Cognitive Impairment: A Systematic Review and Meta-Analysis. J. Alzheimers Dis..

[302] Yong L., Liu L., Ding T., Yang G., Su H., Wang J., Yang M., Chang J. (2021). Evidence of Effect of Aerobic Exercise on Cognitive Intervention in Older Adults with Mild Cognitive Impairment. Front. Psychiatry.

[303] Ngandu T., Lehtisalo J., Solomon A., Levalahti E., Ahtiluoto S., Antikainen R., Backman L., Hanninen T., Jula A., Laatikainen T. (2015). A 2-year multidomain intervention of diet, exercise, cognitive training, and vascular risk monitoring versus control to prevent cognitive decline in at-risk elderly people (FINGER): A randomised controlled trial. Lancet.

[304] Kivipelto M., Mangialasche F., Ngandu T. (2018). Lifestyle interventions to prevent cognitive impairment, dementia and Alzheimer disease. Nat. Rev. Neurol..

[305] Mattson M.P. (2000). Emerging neuroprotective strategies for Alzheimer’s disease: Dietary restriction, telomerase activation, and stem cell therapy. Exp. Gerontol..

[306] Scarmeas N., Stern Y., Tang M.X., Mayeux R., Luchsinger J.A. (2006). Mediterranean diet and risk for Alzheimer’s disease. Ann. Neurol..

[307] Singh B., Parsaik A.K., Mielke M.M., Erwin P.J., Knopman D.S., Petersen R.C., Roberts R.O. (2014). Association of mediterranean diet with mild cognitive impairment and Alzheimer’s disease: A systematic review and meta-analysis. J. Alzheimers Dis..

[308] Brouwer-Brolsma E.M., Benati A., van de Wiel A., van Lee L., de Vries J.H.M., Feskens E.J.M., van de Rest O. (2018). Higher Mediterranean Diet scores are not cross-sectionally associated with better cognitive scores in 20- to 70-year-old Dutch adults: The NQplus study. Nutr. Res..

[309] Karstens A.J., Tussing-Humphreys L., Zhan L., Rajendran N., Cohen J., Dion C., Zhou X.J., Lamar M. (2019). Associations of the Mediterranean diet with cognitive and neuroimaging phenotypes of dementia in healthy older adults. Am. J. Clin. Nutr..

[310] Shannon O.M., Stephan B.C.M., Granic A., Lentjes M., Hayat S., Mulligan A., Brayne C., Khaw K.T., Bundy R., Aldred S. (2019). Mediterranean diet adherence and cognitive function in older UK adults: The European Prospective Investigation into Cancer and Nutrition-Norfolk (EPIC-Norfolk) Study. Am. J. Clin. Nutr..

[311] Omar S.H. (2019). Mediterranean and MIND Diets Containing Olive Biophenols Reduces the Prevalence of Alzheimer’s Disease. Int. J. Mol. Sci..

[312] Gustafson D.R., Backman K., Scarmeas N., Stern Y., Manly J.J., Mayeux R., Gu Y. (2020). Dietary fatty acids and risk of Alzheimer’s disease and related dementias: Observations from the Washington Heights-Hamilton Heights-Inwood Columbia Aging Project (WHICAP). Alzheimers Dement..

[313] Morris M.C., Evans D.A., Bienias J.L., Tangney C.C., Bennett D.A., Wilson R.S., Aggarwal N., Schneider J. (2003). Consumption of fish and n-3 fatty acids and risk of incident Alzheimer disease. Arch. Neurol..

[314] Young G., Conquer J. (2005). Omega-3 fatty acids and neuropsychiatric disorders. Reprod. Nutr. Dev..

[315] Freund-Levi Y., Eriksdotter-Jonhagen M., Cederholm T., Basun H., Faxen-Irving G., Garlind A., Vedin I., Vessby B., Wahlund L.O., Palmblad J. (2006). Omega-3 fatty acid treatment in 174 patients with mild to moderate Alzheimer disease: OmegAD study: A randomized double-blind trial. Arch. Neurol..

[316] Chiu C.C., Su K.P., Cheng T.C., Liu H.C., Chang C.J., Dewey M.E., Stewart R., Huang S.Y. (2008). The effects of omega-3 fatty acids monotherapy in Alzheimer’s disease and mild cognitive impairment: A preliminary randomized double-blind placebo-controlled study. Prog. Neuropsychopharmacol. Biol. Psychiatry.

[317] Bastianetto S., Ramassamy C., Dore S., Christen Y., Poirier J., Quirion R. (2000). The Ginkgo biloba extract (EGb 761) protects hippocampal neurons against cell death induced by beta-amyloid. Eur. J. Neurosci..

[318] Bastianetto S., Yao Z.X., Papadopoulos V., Quirion R. (2006). Neuroprotective effects of green and black teas and their catechin gallate esters against beta-amyloid-induced toxicity. Eur. J. Neurosci..

[319] Hartman R.E., Shah A., Fagan A.M., Schwetye K.E., Parsadanian M., Schulman R.N., Finn M.B., Holtzman D.M. (2006). Pomegranate juice decreases amyloid load and improves behavior in a mouse model of Alzheimer’s disease. Neurobiol. Dis..

[320] Niidome T., Takahashi K., Goto Y., Goh S., Tanaka N., Kamei K., Ichida M., Hara S., Akaike A., Kihara T. (2007). Mulberry leaf extract prevents amyloid beta-peptide fibril formation and neurotoxicity. NeuroReport.

[321] Barnard N.D., Bush A.I., Ceccarelli A., Cooper J., de Jager C.A., Erickson K.I., Fraser G., Kesler S., Levin S.M., Lucey B. (2014). Dietary and lifestyle guidelines for the prevention of Alzheimer’s disease. Neurobiol. Aging.

[322] Hasan-Olive M.M., Lauritzen K.H., Ali M., Rasmussen L.J., Storm-Mathisen J., Bergersen L.H. (2019). A Ketogenic Diet Improves Mitochondrial Biogenesis and Bioenergetics via the PGC1alpha-SIRT3-UCP2 Axis. Neurochem. Res..

[323] McCarty M.F., Di Nicolantonio J.J., O’Keefe J.H. (2015). Ketosis may promote brain macroautophagy by activating Sirt1 and hypoxia-inducible factor-1. Med. Hypotheses.

[324] Elamin M., Ruskin D.N., Masino S.A., Sacchetti P. (2017). Ketone-Based Metabolic Therapy: Is Increased NAD^+^ a Primary Mechanism?. Front. Mol. Neurosci..

[325] McDaniel S.S., Rensing N.R., Thio L.L., Yamada K.A., Wong M. (2011). The ketogenic diet inhibits the mammalian target of rapamycin (mTOR) pathway. Epilepsia.

[326] Kashiwaya Y., Bergman C., Lee J.H., Wan R., King M.T., Mughal M.R., Okun E., Clarke K., Mattson M.P., Veech R.L. (2013). A ketone ester diet exhibits anxiolytic and cognition-sparing properties, and lessens amyloid and tau pathologies in a mouse model of Alzheimer’s disease. Neurobiol. Aging.

[327] Wang R., Li J.J., Diao S., Kwak Y.D., Liu L., Zhi L., Bueler H., Bhat N.R., Williams R.W., Park E.A. (2013). Metabolic stress modulates Alzheimer’s β-secretase gene transcription via SIRT1-PPARγ-PGC-1 in neurons. Cell Metab..

[328] Mota B.C., Sastre M. (2021). The Role of PGC1alpha in Alzheimer’s Disease and Therapeutic Interventions. Int. J. Mol. Sci..

[329] Warren E.C., Dooves S., Lugara E., Damstra-Oddy J., Schaf J., Heine V.M., Walker M.C., Williams R.S.B. (2020). Decanoic acid inhibits mTORC1 activity independent of glucose and insulin signaling. Proc. Natl. Acad. Sci. USA.

[330] Croteau E., Castellano C.A., Richard M.A., Fortier M., Nugent S., Lepage M., Duchesne S., Whittingstall K., Turcotte E.E., Bocti C. (2018). Ketogenic Medium Chain Triglycerides Increase Brain Energy Metabolism in Alzheimer’s Disease. J. Alzheimers Dis..

[331] Croteau E., Castellano C.A., Fortier M., Bocti C., Fulop T., Paquet N., Cunnane S.C. (2018). A cross-sectional comparison of brain glucose and ketone metabolism in cognitively healthy older adults, mild cognitive impairment and early Alzheimer’s disease. Exp. Gerontol..

[332] Wang J., Ho L., Qin W., Rocher A.B., Seror I., Humala N., Maniar K., Dolios G., Wang R., Hof P.R. (2005). Caloric restriction attenuates beta-amyloid neuropathology in a mouse model of Alzheimer’s disease. FASEB J..

[333] Patel N.V., Gordon M.N., Connor K.E., Good R.A., Engelman R.W., Mason J., Morgan D.G., Morgan T.E., Finch C.E. (2005). Caloric restriction attenuates Aβ-deposition in Alzheimer transgenic models. Neurobiol. Aging.

[334] Qin W., Chachich M., Lane M., Roth G., Bryant M., de Cabo R., Ottinger M.A., Mattison J., Ingram D., Gandy S. (2006). Calorie restriction attenuates Alzheimer’s disease type brain amyloidosis in Squirrel monkeys (*Saimiri sciureus*). J. Alzheimers Dis..

[335] Halagappa V.K., Guo Z., Pearson M., Matsuoka Y., Cutler R.G., Laferla F.M., Mattson M.P. (2007). Intermittent fasting and caloric restriction ameliorate age-related behavioral deficits in the triple-transgenic mouse model of Alzheimer’s disease. Neurobiol. Dis..

[336] Mouton P.R., Chachich M.E., Quigley C., Spangler E., Ingram D.K. (2009). Caloric restriction attenuates amyloid deposition in middle-aged dtg APP/PS1 mice. Neurosci. Lett..

[337] Schafer M.J., Alldred M.J., Lee S.H., Calhoun M.E., Petkova E., Mathews P.M., Ginsberg S.D. (2015). Reduction of β-amyloid and γ-secretase by calorie restriction in female Tg2576 mice. Neurobiol. Aging.

[338] Dong W., Wang R., Ma L.N., Xu B.L., Zhang J.S., Zhao Z.W., Wang Y.L., Zhang X. (2016). Influence of age-related learning and memory capacity of mice: Different effects of a high and low caloric diet. Aging Clin. Exp. Res..

[339] Qin W., Yang T., Ho L., Zhao Z., Wang J., Chen L., Zhao W., Thiyagarajan M., MacGrogan D., Rodgers J.T. (2006). Neuronal SIRT1 activation as a novel mechanism underlying the prevention of Alzheimer disease amyloid neuropathology by calorie restriction. J. Biol. Chem..

[340] Muller L., Power Guerra N., Stenzel J., Ruhlmann C., Lindner T., Krause B.J., Vollmar B., Teipel S., Kuhla A. (2021). Long-Term Caloric Restriction Attenuates β-Amyloid Neuropathology and Is Accompanied by Autophagy in APPswe/PS1delta9 Mice. Nutrients.

[341] Van Cauwenberghe C., Vandendriessche C., Libert C., Vandenbroucke R.E. (2016). Caloric restriction: Beneficial effects on brain aging and Alzheimer’s disease. Mamm. Genome.

[342] Yang Y., Zhang L. (2020). The effects of caloric restriction and its mimetics in Alzheimer’s disease through autophagy pathways. Food Funct..

[343] Singh R., Lakhanpal D., Kumar S., Sharma S., Kataria H., Kaur M., Kaur G. (2012). Late-onset intermittent fasting dietary restriction as a potential intervention to retard age-associated brain function impairments in male rats. Age.

[344] Stekovic S., Hofer S.J., Tripolt N., Aon M.A., Royer P., Pein L., Stadler J.T., Pendl T., Prietl B., Url J. (2019). Alternate Day Fasting Improves Physiological and Molecular Markers of Aging in Healthy, Non-obese Humans. Cell Metab..

[345] Dias G.P., Murphy T., Stangl D., Ahmet S., Morisse B., Nix A., Aimone L.J., Aimone J.B., Kuro O.M., Gage F.H. (2021). Intermittent fasting enhances long-term memory consolidation, adult hippocampal neurogenesis, and expression of longevity gene Klotho. Mol. Psychiatry.

[346] Yang P., Sheng D., Guo Q., Wang P., Xu S., Qian K., Li Y., Cheng Y., Wang L., Lu W. (2020). Neuronal mitochondria-targeted micelles relieving oxidative stress for delayed progression of Alzheimer’s disease. Biomaterials.

[347] Toledo J.B., Arnold M., Kastenmuller G., Chang R., Baillie R.A., Han X., Thambisetty M., Tenenbaum J.D., Suhre K., Thompson J.W. (2017). Metabolic network failures in Alzheimer’s disease: A biochemical road map. Alzheimers Dement..

[348] Augustin K., Khabbush A., Williams S., Eaton S., Orford M., Cross J.H., Heales S.J.R., Walker M.C., Williams R.S.B. (2018). Mechanisms of action for the medium-chain triglyceride ketogenic diet in neurological and metabolic disorders. Lancet Neurol..

[349] Garcia-Mesa Y., Colie S., Corpas R., Cristofol R., Comellas F., Nebreda A.R., Gimenez-Llort L., Sanfeliu C. (2016). Oxidative Stress Is a Central Target for Physical Exercise Neuroprotection Against Pathological Brain Aging. J. Gerontol. A Biol. Sci. Med. Sci..

[350] Maliszewska-Cyna E., Lynch M., Oore J.J., Nagy P.M., Aubert I. (2017). The Benefits of Exercise and Metabolic Interventions for the Prevention and Early Treatment of Alzheimer’s Disease. Curr. Alzheimer Res..

[351] Broom G.M., Shaw I.C., Rucklidge J.J. (2019). The ketogenic diet as a potential treatment and prevention strategy for Alzheimer’s disease. Nutrition.

[352] Rusek M., Pluta R., Ulamek-Koziol M., Czuczwar S.J. (2019). Ketogenic Diet in Alzheimer’s Disease. Int. J. Mol. Sci..

[353] Carvalho C., Cardoso S. (2021). Diabetes-Alzheimer’s Disease Link: Targeting Mitochondrial Dysfunction and Redox Imbalance. Antioxid. Redox Signal..

